# Feasibility and evaluation of a large-scale external validation approach for patient-level prediction in an international data network: validation of models predicting stroke in female patients newly diagnosed with atrial fibrillation

**DOI:** 10.1186/s12874-020-00991-3

**Published:** 2020-05-06

**Authors:** Jenna M. Reps, Ross D. Williams, Seng Chan You, Thomas Falconer, Evan Minty, Alison Callahan, Patrick B. Ryan, Rae Woong Park, Hong-Seok Lim, Peter Rijnbeek

**Affiliations:** 1 Janssen Research and Development, 1125 Trenton Harbourton Rd, Titusville, NJ 08560 USA; 2 Department of Medical Informatics, Erasmus University Medical Center, Rotterdam, The Netherlands; 3 Department of Biomedical Informatics, Ajou University School of Medicine, Suwon, Republic of Korea; 4 Department of Biomedical Informatics, Columbia University Medical Center, New York, USA; 5 O’Brien Institute for Public Health, Faculty of Medicine, University of Calgary, Calgary, Alberta Canada; 6 Center for Biomedical Informatics Research, School of Medicine, Stanford University, Stanford, CA USA; 7 Department of Biomedical Sciences, Ajou University Graduate School of Medicine, Suwon, Republic of Korea; 8 Department of Cardiology, Ajou University Medical Centre, Suwon, Republic of Korea

**Keywords:** Patient-level prediction, Prognostic model, External validation, Transportability, Collaborative network

## Abstract

**Background:**

To demonstrate how the Observational Healthcare Data Science and Informatics (OHDSI) collaborative network and standardization can be utilized to scale-up external validation of patient-level prediction models by enabling validation across a large number of heterogeneous observational healthcare datasets.

**Methods:**

Five previously published prognostic models (ATRIA, CHADS_2_, CHADS_2_VASC, Q-Stroke and Framingham) that predict future risk of stroke in patients with atrial fibrillation were replicated using the OHDSI frameworks. A network study was run that enabled the five models to be externally validated across nine observational healthcare datasets spanning three countries and five independent sites.

**Results:**

The five existing models were able to be integrated into the OHDSI framework for patient-level prediction and they obtained mean c-statistics ranging between 0.57–0.63 across the 6 databases with sufficient data to predict stroke within 1 year of initial atrial fibrillation diagnosis for females with atrial fibrillation. This was comparable with existing validation studies. The validation network study was run across nine datasets within 60 days once the models were replicated. An R package for the study was published at https://github.com/OHDSI/StudyProtocolSandbox/tree/master/ExistingStrokeRiskExternalValidation.

**Conclusion:**

This study demonstrates the ability to scale up external validation of patient-level prediction models using a collaboration of researchers and a data standardization that enable models to be readily shared across data sites. External validation is necessary to understand the transportability or reproducibility of a prediction model, but without collaborative approaches it can take three or more years for a model to be validated by one independent researcher. In this paper we show it is possible to both scale-up and speed-up external validation by showing how validation can be done across multiple databases in less than 2 months. We recommend that researchers developing new prediction models use the OHDSI network to externally validate their models.

## Key points

- External validation of patient-level prediction models is necessary for understanding generalizability of models but validation generally takes three or more years [1]
- We demonstrate that the Observational Healthcare Data Science and Informatics (OHDSI) network and standardizations enable external validation to be completed across multiple sites and countries in days
- We hope other researchers will utilize the OHDSI network to externally validate new and existing patient-level prediction models

## Background

Observational healthcare data often contains longitudinal medical records for large heterogeneous populations. There has been increased interest in learning patient-level prediction models using these big real-world datasets with the aim of improving healthcare [2]. These patient-level prediction models can be used to identify high-risk subgroups that could benefit from interventions. For example, the cardiovascular model QRISK2, that was developed using a UK primary care database, is used to identify patients who may benefit from lipid -lowering medication [3]. It is important to ensure a model has good performance before it is used clinically and this requires external validation [2, 4].

Models are often internally validated using the development dataset by withholding a subset of that data from the model training stage so that it can be used for evaluating the model performance. The majority of patient-level prediction models will report internal validation. External validation is accomplished by evaluating the model on a new dataset (that is different from the development dataset). Few published patient-level prediction models are externally validated, and research has shown that it often takes three or more years for external validation to occur once a model is published [1].

External validation of a patient-level prediction model can provide useful insights into the accuracy of the model across different patient characteristics and may be used to learn the impact of missing predictors. The type of external validation depends on the similarity between the development and validation datasets. When a model is validated on a population that has similar characteristics to the development data population the ‘generalizability performance’ of the model is investigated (i.e., how well the model performs when making predictions on similar patients). When a model is validated on a population that has different characteristics to the development data population the ‘transportability performance’ of the model is investigated (i.e., how well the model performs on different patients). Many observational datasets are not representative of the whole population, so the transportability performance of the model discovered during external validation on patients with different characteristics is important to know when identifying who the model can be broadly applied to. For example, some clinical guidelines recommend treatment stratification for patients based on applying a simple risk score model that was developed on a small population but the transportability of the model to the general population may not have been studied. This may lead to incorrect predictions.

External validation is a slow process due to the difficulty finding suitable data to replicate a prediction model on and difficulty replicating a prediction model (e.g., writing code to correctly extract the same model covariates from the new data). Published papers often lack the information required to replicate the model or can be interpreted subjectively (e.g., in defining medical conditions or variables) which can be an issue causing models to be replicated incorrectly. This prevents efficient and large-scale external validation which likely slows down clinical uptake of published patient-level prediction models or results in the models being applied clinically to patient populations where the model transportability is unknown.

A collaborative approach to external model validation has been proposed to enable extensive evaluation of prediction models [5]. The Observational Healthcare Data Science and Informatics (OHDSI) network is a community of researchers that are working towards the common goal of improving the analysis of observational data [6]. The OHDSI community have developed standardizations that enable efficient collaboration across research sites. The main standardization is the common data structure and vocabulary used by all collaborators known as the Observational Medical Outcomes Partnership (OMOP) common data model. The OMOP common data model ensures all researchers have their data in the same structure so analysis codes such as Structured Query Language (SQL) can be shared across sites. This has enabled the development of analysis packages in R for causal inference and patient-level prediction [7] that can be used by any researcher with data in the OMOP common data model. The OHDSI collaborative network, common data model and patient-level prediction package now present the opportunity to scale up external validation.

The aim of this study is to demonstrate that the OHDSI tools and OMOP common data model can be used by researchers to investigate the external validation performance of their prediction models across a large number of heterogeneous patient populations. Instead of taking years to externally validate a model, OHDSI may make it possible to apply a prediction models to a large number of datasets in a short period of time. To demonstrate this we selected the prediction problem of 1-year risk of stroke in newly diagnosed atrial fibrillation patients as there are multiple existing models that are used clinically, namely Anticoagulation and Risk Factors in Atrial Fibrillation (ATRIA) (no prior stroke model) [8], Framingham (no prior stroke model) [9], Congestive heart failure, Hypertension, Age > 75, Diabetes, prior Stroke/transient ischemic attack (CHADS_2_) [10], CHADS_2_-VASc [11] and Q-Stroke (female model) [12]. We show these models can be replicated using the OHDSI standardizations and externally validated across numerous data sites within the OHDSI network.

## Methods

### Existing stroke prediction models

We selected the problem of predicting stroke in patients with atrial fibrillation as it has been well studied and is one of the only prediction problems to have been extensively validated. Therefore, we have ample benchmarks to compare to the results of this study. The existing models we replicated were ATRIA, CHADS_2_, CHA_2_DS_2−_VASc, Framingham and Q-Stroke.

The ATRIA [8] model was developed on a cohort of 7284 patients who were 18+ and had an atrial fibrillation outpatient diagnosis during 1997 or 1998. ATRIA was internally validated on a 3643 patient hold out set obtaining a c-statistic of 0.72. In the same paper, the authors also externally validated the model on a cohort of 33,247 patients aged 21+ with inpatient or outpatient atrial fib or flutter during 2006–2009, obtaining a c-statistic of 0.7. The CHADS_2_ score [10] was developed by combining two other stroke prediction models (using the variables from these models and assigning points) and was validated on 1733 patients aged 65 to 95 years who had nonrheumatic atrial fibrillation. The CHADS_2_ score obtained a c-statistic of 0.81 on this population. The CHA_2_DS_2−_VASc score [11] is another score-based model that was developed using knowledge of risk factors. The model was validated on a cohort of 1577 patients who were 18+ and had atrial fibrillation during 2003 to 2004 from 35 countries. The model obtained a c-statistic of 0.61 for this patient population. The Framingham score [9] model was based on a Cox model developed using data from 705 patients aged 55 to 94 with initial atrial fibrillation. The internal validation, using a bootstrap approach, showed a c-statistic of 0.66. The Q-Stroke [12] model was developed using primary care data from the UK consisting of 3, 549, 478 patients aged 25–84 with no prior stroke or anticoagulation use (except aspirin) and was internally validated on 1, 897, 168 similar patients. When applying the model to predict the 10-year risk of stroke in female patients with atrial fibrillation at baseline, the c-statistic was 0.65.

The existing models include a small number of variables, Table 1 summarizes the variables included in each model. Some of the variables are unlikely to be available in claims data and these are marked with the + symbol. A large number of Q-Stroke variables are not commonly recorded in claims data (or are UK specific), so this model is difficult to replicate in external non-UK databases. For example, US claims data contain incomplete measurement records and rarely record family history but many of the Q-stroke predictors were recent measurements or family history. Table 2 presents the internal performance and published external validation performance for the five models. Although the internal validation c-statistic for some of the models was as high as 0.8, independent external validation studies of the models tend to show the models achieve c-statistics between 0.6 and 0.7.

**Table 1:** The covariates included in ATRIA, Framingham, CHADS_2,_ CHA_2_DS_2_VASc and Q-Stroke

**Predictor**	**ATRIA**	**Framingham**	**CHADS**_**2**_	**CHA**_**2**_**DS**_**2**_**VASc**	**Q-Stroke**
Age >= 85	x				
Age 75–84	x				
Age 65–74	x			x	
Age 60–62		x			
Age 63–66		x			
Age 67–71		x			
Age 72–74		x			
Age 75–77		x			
Age 78–81		x			
Age 82–85		x			
Age 86–90		x			
Age 91–93		x			
Age > 93		x			
Age >= 75			x	x	
Female	x	x		x	
Diabetes	x	x	x	x	x
Congestive heart failure	x		x		x
Prior Stroke or transient ischemic attack		x	x	x	
Hypertension	x		x	x	x
Systolic blood pressure^+^		x			x
Total cholesterol: HDL^a^ cholesterol ratio^+^					x
Townsend deprivation score^+^					x
Proteinuria	x				
eGFR^a^ < 45 or End stage renal disease	x				
Vascular disease				x	
Congestive heart failure or Liver disease				x	
Smoking status^+^					x
Ethnicity^+^					x
Coronary heart disease					x
Family history of congestive heart failure^+^					x
Atrial fibrillation					x
Rheumatoid arthritis					x
Chronic renal disease					x
Valvular heart disease					x

Existing models for predicting stroke risk. ^+^ indicates predictors are often poorly recorded or missing in claims data

^a^*HDL* High-density lipoproteins, *eGFR* Estimated glomerular filtration rate

**Table 2:** The internal and external validation performances of the existing stroke prediction models

	**ATRIA**	**Framingham**	**CHADS**_**2**_	**CHA**_**2**_**DS**_**2**_**VASc**	**Q-Stroke**
**Internal c-statistic**	0.72	0.66	0.82	0.61	0.65
**External c-statistic**					
UK Electronic Medical Records (EMR) 2015 [13]	0.7 (0.69–0.71)	–	0.68 (0.67–0.69)	0.68 (0.67–0.69)	–
Swedish EMR 2016 [14]	0.71 (0.70–0.71)	–	0.69 (0.69–0.70)	0.69 (0.69–0.70)	–
Taiwan 2016 [15]	–	–	0.66	0.70	–
New Zealand, Russia and the Netherlands 2014 [16]	–	0.70 (0.68–0.73)	–	–	0.71 (0.69–0.73)
UK EMR 2010 [17]	–	0.65 (0.63–0.68)	0.66 (0.64–0.68)	0.67 (0.65–0.69)	–

Internal and previously published external model fit statistics for each of the five models that predict stroke in atrial fibrillation patients

The complete definitions for each variable (sets of SNOMED CT or RXNorm codes) are provided in Additional file 1.

### Validation prediction task

Within a target population of female patients with newly diagnosed atrial fibrillation and no prior stroke predict who will develop a stroke 1 to 365 days after initial diagnosis of atrial fibrillation.

### Sources of data

We validated the existing models using a retrospective cohort design and various observational healthcare datasets (e.g., claims data and electronic healthcare data). The datasets used to evaluate the models are:

IBM MarketScan® Commercial Database (CCAE) is a United States employer-sponsored insurance health plans claims database. The database contains claims (e.g. inpatient, outpatient, and outpatient pharmacy) from private healthcare coverage to employees, their spouses, and dependents, so patients are aged 65 or younger. The database contains data collected between 2000 and 2018.

IBM MarketScan® Medicare Supplemental Database (MDCR) represents health services of retirees in the United States with primary or Medicare supplemental coverage through privately insured fee-for-service, point-of-service, or capitated health plans. The patients are aged 65 or older. The database contains data collected between 2000 and 2018.

IBM MarketScan® Multi-State Medicaid Database (MDCD) contains adjudicated US health insurance claims for Medicaid enrollees from multiple states and includes hospital discharge diagnoses, outpatient diagnoses and procedures, and outpatient pharmacy claims as well as ethnicity. The database contains data collected between 2006 and 2018.

Optum© De-Identified Clinformatics® Data Mart Database – Socio-Economic Status (Optum Claims) is an adjudicated administrative health claims database for members with private health insurance. The population is primarily representative of US commercial claims patients (0–65 years old) with some Medicare (65+ years old) however ages are capped at 90 years. The database contains data collected between 2000 and 2018.

Optum© de-identified Electronic Health Record Dataset (Optum EHR) is a US electron health record containing clinical information, inclusive of prescriptions as prescribed and administered, lab results, vital signs, body measurements, diagnoses, procedures, and information derived from clinical Notes using Natural Language Processing (NLP). The database contains data collected between 2006 and 2018.

Stanford Translational Research Integrated Database Environment (STRIDE) is a clinical data warehouse that supports clinical and translational research at Stanford University. This resource includes the EHR data of approximately 2 million adult and pediatric patients cared for at either the Stanford Hospital or the Lucile Packard Children’s hospital. This study was completed on an OMOP-CDM adherent instance of STRIDE. The database contains data collected between 2000 and 2018.

Columbia University Medical Center’s (CUMC) data come from New York Presbyterian hospital’s clinical data warehouse. The database comprises EHR data on approximately 5 million patients and includes information such as diagnoses, procedures, lab measurements and prescriptions. The database contains data collected between 1980 and 2018.

Ajou University School Of Medicine (AUSOM) is a database containing the entire EHR data from 1994 to 2018 of Korean tertiary hospital, Ajou university hospital. It contains medical record of about 2.9 million patients. The database contains data collected between 1994 and 2018.

The Integrated Primary Care Information (IPCI) is an electronic health care database containing patients of Dutch general practitioners (primary care). The database contains data collected between 1996 and 2018.

Each site had institutional review board approval for the analysis, or used deidentified data and thus the analysis was determined not to be human subjects research and informed consent was not deemed necessary at any site.

### Participants

The existing models were applied to two target populations. Both target populations consisted of female patients newly diagnosed with atrial fibrillation and no prior stroke or anticoagulant use but target population 1 was patients aged 65 to 95 and target population 2 was all ages.

Target population 1: The target populations was defined as females aged 65–95 with either:

- 2 atrial fibrillation records
- 1 atrial fibrillation in an inpatient setting
- 1 atrial fibrillation with an electrocardiogram (ECG) within 30 days prior

and at least 730 days prior database observation and no prior stroke and no prior anticoagulant.

Target population 2: The target populations was defined as females with either:

- 2 atrial fibrillation records
- 1 atrial fibrillation in an inpatient setting
- 1 atrial fibrillation with an ECG within 30 days prior

and at least 730 days prior database observation and no prior stroke and no prior anticoagulant.

The target populations may contain different types of patients per database (e.g., different country US, European or Asian patients and different types of records such as inpatient and outpatient). The different databases used in this study are detailed in section ‘Sources of data’.

### Outcome

We predicted stroke occurring 1 day until 365 days after the initial atrial fibrillation start date. The stroke outcome was defined as:

- An ischemic or hemorrhagic stroke recorded with an inpatient or ER visit

The code sets used to define atrial fibrillation, ECG and ischemic or hemorrhagic stroke are presented in Additional file 2. The full analysis code (data creation and model evaluation) is available at: https://github.com/OHDSI/StudyProtocolSandbox/tree/master/ExistingStrokeRiskExternalValidation

#### Sensitivity analysis

Patients with a high risk of future stroke are often given anticoagulants as a preventative. If a high-risk patient is given an anticoagulant intervention during the 1-year time-at-risk this may prevent the stroke. We therefore performed a sensitivity analysis to remove patients who had an anticoagulant during the 1-year time-at-risk that may have prevented a stroke. For the sensitivity analysis, the target populations were modified by censoring patients at the point an anticoagulant was recorded, so any patient with an anticoagulant during the time-at-risk period was effectively removed from the target population unless they had a stroke prior to the anticoagulant.

### Predictors

We calculated existing model predictors using phenotype definitions specified in the paper describing the development of the model when provided. If the development paper did not provide a definition, we used our own. The definitions for each predictor can be found in Additional file 1.

#### Missing data

Age and gender are required by the OMOP common data model used by OHDSI and will never be missing.

For each condition (diabetes, chronic heart failure, stroke, hypertension, proteinuria, end stage renal disease (ESRD), vascular disease, liver disease, coronary heart disease (CHD), atrial fibrillation, rheumatoid arthritis, chronic renal disease and valvular heart disease), we considered no records of the condition in the database to mean the patient does not have the condition. Ethnicity is often missing completely from a database and when missing we did not include it. Smoking status and family history are rarely recorded in claims data, we imputed 0 (never smoker and no family history) when the predictor was missing. Townsend deprivation score is specific to the UK and was not included as a predictor in our validation. The blood pressure and cholesterol measurements are rarely recorded in claims data and were not included as predictors in our validation.

### Statistical analysis

The prediction model performances were evaluated using the area under the receiver operating characteristic (AUROC) curve which is equivalent to the c-statistic for binary classification. Confidence intervals were also calculated when the number of outcome patients was fewer than 1000. As the models are being used to predict 1-year risk in diverse patients we recalibrated the models for each database. The models were recalibrated by fitting a linear model to the predicted scores to learn a database specific intercept and gradient. We present the calibration plots for each of the five models recalibrated in each of the datasets. For each decile we calculate the mean recalibrated predicted risk and plot against the observed fraction of patients who have the outcome.

### Development vs validation

We picked participants that matched all eligibility criteria for all 5 existing models being validated but this may be a subset of the patient population used to develop the model for many of the models. Many of the predictors for the Q-stroke model were not available in our data and the measurements for Framingham were also no available. The outcome in this validation study was 1 year following index but many of the models were developed for 10-year risk.

## Results

### Participants

The characteristics of the participants across the network showed that hypertension was very common in the patients. Patients were older and often has renal and cardiac issues. See Additional file 3 for the full characteristic table.

IPCI did not contain inpatient stroke records, so the models were unable to be evaluated on this dataset. The percentage of patients who had stroke recorded within 1 year in each of the remaining dataset target populations is presented in Table 3. The percentage of patients with stroke during the 1 year following atrial fibrillation diagnosis in the various target populations ranged from approximately 1% in CCAE, STRIDE, AUSOM and Optum EHR to 5% in MDCD and CUMC.

**Table 3:** The stroke rate (% of target population) across the datasets

	Outcome rate % (Target population size)							
Target Population	CCAE	MDCD	MDCR	Optum claims	Optum EHR	CUMC	AUSOM	STRIDE
T1: Females aged 65+ with atrial fibrillation no prior stroke or anticoagulants	–	4.95 (25,880)	4.40 (89,156)	4.07 (110,905)	1.30 (149,906)	5.75 (4312)	2.61 (268)	1.37 (3366)
T2: Females with atrial fibrillation no prior stroke or anticoagulants	1.33 (61,224)	4.61 (33,262)	–	3.49 (139,376)	1.13 (189,815)	5.00 (5758)	1.76 (455)	1.28 (4456)
Sensitivity T1: Females aged 65+ with atrial fibrillation no prior stroke or anticoagulants (no anticoagulants during tar)	–	5.04 (23,586)	5.26 (56,511)	4.48 (78,353)	1.44 (99,212)	6.23 (3403)	4.17 (144)	1.29 (2094)
Sensitivity T2: Females with atrial fibrillation no prior stroke or anticoagulants (no anticoagulants during tar)	1.28 (46,054)	4.69 (29,546)	–	3.73 (100,757)	1.22 (128,409)	5.35 (4546)	2.73 (256)	1.22 (2786)

Target population size in each dataset and the percentage of patients with stroke within 1 year of initial atrial fibrillation diagnosis

### Model performance

The results of the discriminative ability of the five existing models across all eight datasets that had inpatient stroke recorded are presented in Table 4. As the AUSOM and STRIDE datasets had outcome counts less than 100, we report the performance in Table 4 but do not include it in the aggregate summaries due to uncertainty in the estimates as a result of small sample sizes.

**Table 4:** Discrimination performance of the existing models externally validated across the OHDSI datasets

		Database AUROC (95% CIs)							
Target Population^a^	Model	CCAE	MDCD	MDCR	Optum claims	Optum EHR	CUMC	AUSOM	STRIDE
T1: Females aged 65+ with atrial fibrillation no prior stroke or anticoagulants	ATRIA	–	0.57 (0.55–0.58)	0.63 (0.62–0.64)	0.61	0.62	0.64 (0.61–0.68)	0.60 (0.33–0.87)	0.49 (0.40–0.58)
	CHADS_2_	–	0.54 (0.53–0.56)	0.60 (0.59–0.61)	0.59	0.60	0.60 (0.57–0.64)	0.51 (0.27–0.75)	0.48 (0.39–0.57)
	CHA_2_DS_2_VASc	–	0.55 (0.53–0.57)	0.60 (0.59–0.61)	0.59	0.62	0.61 (0.58–0.65)	0.53 (0.32–0.74)	0.52 (0.42–0.62)
	Framingham	–	0.56 (0.54–0.57)	0.62 (0.61–0.63)	0.59	0.61	0.63 (0.60–0.66)	0.58 (0.33–0.83)	0.61 (0.52–0.70)
	Q-Stroke	–	0.53 (0.52–0.55)	0.56 (0.55–0.57)	0.55	0.56	0.55 (0.51–0.59)	0.56 (0.29–0.84)	0.50 (0.41–0.59)
T2: Females with atrial fibrillation no prior stroke or anticoagulants	ATRIA	0.62 (0.60–0.64)	0.58 (0.56–0.59)	–	0.65	0.65	0.66 (0.62–0.69)	0.73 (0.58–0.89)	0.52 (0.44–0.60)
	CHADS_2_	0.61 (0.59–0.62)	0.56 (0.55–0.57)	–	0.62	0.63	0.63 (0.60–0.66)	0.63 (0.43–0.83)	0.50 (0.42–0.57)
	CHA_2_DS_2_VASc	0.63 (0.61–0.65)	0.58 (0.56–0.59)	–	0.64	0.65	0.64 (0.61–0.67)	0.73 (0.60–0.85)	0.55 (0.47–0.62)
	Framingham	0.62 (0.60–0.64)	0.57 (0.56–0.59)	–	0.64	0.65	0.65 (0.62–0.68)	0.70 (0.53–0.86)	0.61 (0.53–0.69)
	Q-Stroke	0.61 (0.59–0.63)	0.54 (0.53–0.56)	–	0.57	0.58	0.56 (0.53–0.60)	0.63 (0.39–0.88)	0.51 (0.43–0.59)
Sensitivity T1: Females aged 65+ with atrial fibrillation no prior stroke or anticoagulants (no anticoagulants during 1 year time-at-risk)	ATRIA	–	0.56 (0.55–0.58)	0.63 (0.62–0.64)	0.61 (0.61–0.62)	0.63 (0.61–0.64)	0.65 (0.62–0.69)	0.69 (0.43–0.95)	0.55 (0.47–0.62)
	CHADS_2_	–	0.54 (0.53–0.56)	0.61 (0.60–0.62)	0.59 (0.58–0.60)	0.61 (0.59–0.62)	0.62 (0.58–0.65)	0.61 (0.36–0.85)	0.51 (0.38–0.63)
	CHA_2_DS_2_VASc	–	0.55 (0.54–0.57)	0.61 (0.60–0.62)	0.59 (0.58–0.60)	0.63 (0.61–0.64)	0.63 (0.59–0.66)	0.64 (0.45–0.83)	0.55 (0.42–0.67)
	Framingham	–	0.55 (0.54–0.57)	0.62 (0.61–0.63)	0.59 (0.59–0.60)	0.62 (0.61–0.63)	0.64 (0.61–0.68)	0.68 (0.44–0.93)	0.64 (0.53–0.74)
	Q-Stroke	–	0.53 (0.52–0.55)	0.57 (0.55–0.58)	0.55 (0.54–0.56)	0.57 (0.55–0.58)	0.56 (0.52–0.60)	0.61 (0.30–0.92)	0.47 (0.35–0.58)
Sensitivity T2: Females with atrial fibrillation no prior stroke or anticoagulants (no anticoagulants during 1-year time-at-risk)	ATRIA	0.63 (0.61–0.66)	0.58 (0.56–0.59)	–	0.67	0.67	0.67 (0.64–0.70)	0.79 (0.63–0.94)	0.53 (0.43–0.63)
	CHADS_2_	0.62 (0.60–0.65)	0.56 (0.55–0.58)	–	0.64	0.65	0.64 (0.61–0.68)	0.72 (0.53–0.91)	0.51 (0.41–0.62)
	CHA_2_DS_2_VASc	0.65 (0.62–0.67)	0.58 (0.56–0.59)	–	0.65	0.67	0.66 (0.63–0.69)	0.81 (0.71–0.90)	0.55 (0.44–0.65)
	Framingham	0.64 (0.61–0.66)	0.57 (0.56–0.59)	–	0.65	0.66	0.66 (0.63–0.69)	0.76 (0.59–0.93)	0.62 (0.51–0.72)
	Q-Stroke	0.62 (0.60–0.64)	0.55 (0.53–0.56)	–	0.58	0.6	0.57 (0.53–0.61)	0.68 (0.42–0.94)	0.47 (0.36–0.57)

Discrimination performance of the existing models across the datasets. The AUROC 95% confidence intervals were only calculated when the outcome count was less than 1000. ^a^ See section ‘Participants’ for full inclusion/exclusion criteria

Across the datasets with sufficient outcome counts, ATRIA obtained a mean AUROC of 0.61 (range 0.57–0.64) on the female patients aged 65 or older and a mean AUROC of 0.63 (range 0.58–0.66) on the female patients of all ages. CHADS_2_ obtained a mean AUROC of 0.58 (range 0.54–0.60) on the female patients aged 65 or older and a mean AUROC of 0.61 (range 0.56–0.63) on the female patients of all ages. CHA_2_DS_2_VASc obtained a mean AUROC of 0.60 (range 0.55–0.62) on the female patients aged 65 or older and a mean AUROC of 0.63 (range 0.58–0.65) on the female patients of all ages. Framingham obtained a mean AUROC of 0.60 (range 0.56–0.63) on the female patients aged 65 or older and a mean AUROC of 0.64 (range 0.57–0.65) on the female patients of all ages. Q-Stroke obtained a mean AUROC of 0.55 (range 0.53–0.56) on the female patients aged 65 or older and a mean AUROC of 0.57 (range 0.54–0.61) on the female patients of all ages.

The calibration plots showed that recalibrating the total scores using a linear model appears to work for ATRIA, Q-stroke, CHADS_2_ and CHA_2_DS_2_VASc but the Framingham model may need a non-linear recalibration as it appeared to under-estimate risk in the middle risk groups, see Additional file 4.

## Discussion

This study demonstrated the ability to perform external validation across five different data sites with access to nine databases in a short period of time. The countries corresponding to each database spanned across the USA, Europe and Asia. This shows the OHDSI network and tools can be used by researchers to efficiently perform external validation of models developed using observational healthcare data. The datasets used for validating the existing models that predict stroke in female patients with atrial fibrillation had varied outcome rates (1–6%) indicating differences between the data. Despite the differences between the datasets there was consistently moderate discriminative performance across the databases.

### Interpretation

Excluding patients with an anticoagulant after atrial fibrillation who did not have a prior stroke increased the incidence rate for all databases except CCAE and STRIDE. This suggests many people under 65 who have a stroke within a year of initial atrial fibrillation diagnosis had a prior anticoagulant. This may be a consequence of different treatment of patients with atrial fibrillation who are under 65 compared to being 65 and older. Atrial fibrillation patients who are given an anticoagulant when they are younger than 65 may have other risk factors prompting the use of an anticoagulant.

The sensitivity analysis shows the AUROC performance of models when removing patients with an anticoagulant and no stroke or an anticoagulant prior to stroke is comparable or better, see Table 3. This makes sense, for example consider the hypothetical situation where a clinical risk model correctly assigns a high risk to a patient who will have a stroke, but this high risk leads to a clinician giving the patient anticoagulants before the stroke that prevent the stroke occurring. In this situation the model’s performance will be negatively impacted because of the intervention as the model was correct to assign a high risk but was wrong due to the intervention preventing the stroke. This raises the issue of how to fairly evaluate models that are already being used clinically or in situations where existing guidelines are used to identify patients who should being given preventative medicine. A fair evaluation is simple when there is no clinical intervention, but complex when preventative medicine exists for the outcome.

The validation performance of the models replicated using the OHDSI patient-level prediction framework and validated across the OHDSI network are comparable with other published results. The Q-Stroke model performed the worst out of all the existing models, but this is likely due to many variables of that model being specific to the UK or are things that are missing from claims data (such as family history, smoking status and recent measurements). This may indicate that Q-Stroke is not transportable to the US population. In addition, the performances of the models were worse when applied to older females as age is a key predictor in many of the models. In future work it would be interesting to investigate applying more complex machine learning methods with data-driven predictor selection to learn more advanced models for predicting stroke in older patients with atrial fibrillation and no prior stroke.

### Implications

The external validation was performed over 60 days by five different research sites. Utilizing the OHDSI collaboration to validate a new prognostic model would enable extensive external validation across diverse patient populations. In addition, this could be accomplished in significantly less time than the current process for external validation that takes more than 3 years on average for one other researcher to implement the model [1]. The large-scale external validation was only possible because i) the OMOP common data model and OHDSI standardizations enable sharing of analysis code and ii) collaboration that is possible due to the OHDSI network. We recommend researchers who develop prediction models gain insight into their model’s transportability by utilizing the OHDSI network’s external validation ability. All that is required is to replicate their models using the OHDSI Patient-level prediction framework, which would also enable other researchers to readily implement the model.

### Limitations

The main limitation of this study was the correct replication of existing models. The reason external validation rarely occurs is that many published models lack certain details such as how to define variables, as code lists are often not published. As a best practice patient-level prediction models should provide full definitions for all variables in the model and provide the model. We used the model’s variable definitions when published, but when these were not available, we used our own code sets to define the variables. Another limitation in this study is the limited target populations investigated. We chose females aged 65 or older with no prior stroke as that was the intersection of criteria used when developing the five existing stroke models but we also wanted to see the impact of restricting to older patients (as many models use age as a variable), so we included a second target population of all females with no prior stroke. In future work it would be interesting to investigate the performances of the models across many different target populations. Finally, although OHDSI contains a large network of databases, it may not be possible to validate every prediction model on each of the databases within the network. For example, some databases may not contain the criteria used to identify the target population (e.g., if the target population required a specific measurement), may not have certain predictors recorded or may not have the outcome recorded (e.g., if the outcome requires an inpatient record but the data only contain outpatient records). The databases may also have insufficient observation time (e.g., a model predicting 10-year risk of stroke may not be suitably evaluated in US claims data such as Optum claims where only 13% of patients have 5+ years of observation). Future work needs to be done to investigate how to interpret the results of external validation across heterogeneous datasets.

## Conclusion

In this paper we demonstrated the ability to scale-up external validation by using a collaborative network where researchers share a common data structure. The existing prediction models were validated on 9 databases across 5 sites within 2 months. We recommend that researchers utilize the OHDSI network to externally validate their models at scale across multiple datasets to gain insight into the generalizability and/or transportability of their models.

In addition, the results show that the existing stroke in atrial fibrillation models do not perform well at predicting stroke in the target population of older females in datasets we investigated. This prompts further research into whether a better model can be developed.

## Supplementary information

**Additional file 1. Appendix A.** Variable definitions.

**Additional file 2. Appendix B.** Snomed codes defining atrial fibrillation.

**Additional file 3. Appendix C.** Characterization of patients.

**Additional file 4. Appendix D.** Calibration plots for the females of any age target population.

## Data Availability

The Optum, IBM CCAE, IBM MDCR, and IBM MDCD data that support the findings of this study are available from IBM MarketScan Research Databases (contact at: http://www.ibm.com/us-en/marketplace/marketscan-research-databases) and Optum (contact at: http://www.optum.com/solutions/data-analytics/data/real-world-data-analytics-a-cpl/claims-data.html) but restrictions apply to the availability of these data, which were used under license for the current study, and so are not publicly available. Due to ethical concerns, supporting data cannot be made openly available for the CUMC, STRIDE and AUSOM datasets.

## Abbreviations

ATRIA: Anticoagulation and Risk Factors in Atrial Fibrillation
AUROC: Area under the receiver operating characteristic
AUSOM: Ajou University School Of Medicine
CCAE: IBM MarketScan® Commercial Database
CHADS_2_: Congestive heart failure, Hypertension, Age > 75, Diabetes, prior Stroke/transient ischemic attack
CHD: Coronary heart disease
CUMC: Columbia University Medical Center’s
ECG: Electrocardiogram
*eGFR*: *Estimated glomerular filtration rate*
EMR: Electronic medical records
ESRD: End stage renal disease
*HDL*: *High-density lipoproteins*
IPCI: The Integrated Primary Care Information
MDCD: IBM MarketScan® Multi-State Medicaid Database
MDCR: IBM MarketScan® Medicare Supplemental Database
OHDSI: Observational Healthcare Data Science and Informatics
OMOP: Observational Medical Outcomes Partnership
Optum Claims: Optum© De-Identified Clinformatics® Data Mart Database – Socio-Economic Status
Optum EHR: Optum© de-identified Electronic Health Record Dataset
STRIDE: Stanford Translational Research Integrated Database Environment
SQL: Structured Query Language
