# Supplementary material for: Feasibility and evaluation of a large-scale external validation approach for patient-level prediction in an international data network: validation of models predicting stroke in female patients newly diagnosed with atrial fibrillation
Source: BMC Med Res Methodol. 2020 May 6;20:102. doi: 10.1186/s12874-020-00991-3 (PMC7201646; doi:10.1186/s12874-020-00991-3)
Supplement: Supplementary file 1 — Additional file 1. Appendix A. Variable definitions. [file 12874_2020_991_MOESM1_ESM.docx]

**APPENDIX A – Variable definitions**

| **Variable** | **Snomed concept ids** | **ICD10CM mapped** | **ICD9CM mapped** | **Read mapped** |
| --- | --- | --- | --- | --- |
| Stroke or transient ischemic attack | 4110192, 45767658, 45772786, 4108356, 4110190, 4110189, 4043731, 374060, 373503, 374055, 4338523, 4112020, 4110194, 4108357, 4110195, 4108360, 4043734, 4048785, 40485430, 46272244, 4139517, 36712813, 36712812 | I63.349, I63.19, I63.49, I63.441, I63.231, I63.011, I63.00, I63.429, I63.332, I63.131, I63.30, I63.412, I63.341, I63.439, I63.12, I63.031, I63.419, I63.39, I63.331, I63.322, I63.139, I63.449, I63.132, I63.339, I63.312, I63.10, I63.411, I63.232, I63.119, I63.032, I63.019, I63.321, I63.311, I63.112, I63.442, I63.422, I63.40, I63.20, I63.02, I63.432, I63.431, I63.319, I63.29, I63.421, I63.342, I63.329, I63.239, I63.111, I63.039, I63.09, I63.012, I63.443, I63.433, I63.423, I63.413, I63.343, I63.333, I63.323, I63.313, I63.233, I63.133, I63.113, I63.033, I63.013, G46.2, G46.1, G46.0, G45.9, G45.8, G45.3, G45.1, G45.0, I63.44, I63.43, I63.42, I63.41, I63.4, I63.34, I63.33, I63.32, I63.31, I63.3, I63.23, I63.2, I63.13, I63.11, I63.1, I63.03, I63.01, I63.0, G45 | 434.91, 434.01, 434.11, 433.11, 433.21, 433.31, 433.81, 433.91, 436, 435, 435.0, 435.8, 435.9 | G640000, G641000, G63..11, G63y000, G63y100, F423600, Fyu5500, G65..00, G65..12, G65..13 ,G650.00, G650.11, G653.00, G654.00, G656.00, G657.00, G65y.00, G65z.00, G65zz00, G660.00, G661.00, G662.00, |
| Diabetes | 46274096, 45766052, 45766051, 45766050, 45757789, 45757674, 45757474, 45757129, 45757124, 45757079, 45757077, 44793114, 44793113, 44792134, 44787902, 43531645, 43531644, 43531643, 43531642, 43531641, 43531640, 43531020, 43531019, 43531018, 43531017, 43531016, 43531015, 43531014, 43531013, 43531012, 43531011, 43531010, 43531009, 43531008, 43531007, 43531006, 42538715, 42537681, 42536605, 42536604, 42535539, 40482883, 37396524, 37117810, 37117219, 37116960, 37116379, 37110117, 37110068, 37110041, 37109305, 37018765, 37018566, 36717215, 36716258, 36715417, 36715051, 36714116, 36713275, 4327944, 4326434, 4322638, 4321756, 4304377, 4263902, 4252384, 4245270, 4240589, 4237068, 4235410, 4230254, 4212631, 4202383, 4200875, 4198296, 4196141, 4193704, 4192852, 4178790, 4178452, 4166381, 4152858, 4151281, 4145827, 4144583, 4143529, 4140808, 4136889, 4131907, 4130166, 4130164, 4130162, 4129524, 4129519, 4129516, 4129378, 4102018, 4099741, 4099653, 4099652, 4099651, 4099334, 4099217, 4099215, 4099214, 4096671, 4096670, 4096668, 4096042, 4096041, 4084643, 4079850, 4063043, 4063042, 4062687, 4062686, 4062685, 4058243, 4048202, 4047906, 4034962, 4034960, 4030061, 4024659, 4008576, 4006979, 765478, 443412, 443012, 201826, 201820, 201531, 201530, 201254, 195771, 194700, 193323, 192691 | O24.32, O24.119, O24.02, E11.65, E10.621, E09.638, E08.8, E08.618, E08.44, E08.40, O24.813, E13.69, E11.622, E11.610, E10.649, E10.10, E09.52, E08.329, E08.321, O24.911, E13.65, E11.69, E11.22, E08.65, E08.51, E08.49, E08.43, O24.83, O24.439, E11.649, E09.618, E09.49, E08.351, O24.92, O24.319, O24.12, O24.111, O24.012, E13.610, E11.618, E11.01, E09.8, E09.359, E09.341, E09.11, E08.00, O24.434, O24.429, O24.414, O24.13, O24.112, E13.630, E13.618, E11.621, E10.622, E09.649, E09.51, E09.41, E09.349, E08.621, E08.59, E08.341, O24.812, O24.03, E10.610, E09.621, E09.321, E09.319, E09.311, E09.29, E09.01, O24.919, O24.430, E09.44, E08.9, E08.630, E08.628, E08.10, O24.912, O24.113, E13.638, E13.621, E13.10, E11.638, E09.620, E09.59, E09.339, E08.36, O24.93, O24.811, O24.424, O24.311, E13.00, E11.641, E09.39, E09.22, E09.00, E08.649, E08.52, E08.41, E08.359, O24.419, O24.312, E13.622, E10.630, E09.65, E09.622, E08.01, O24.819, O24.410, E09.641, E09.329, E09.21, E08.641, E08.39, E08.319, E08.22, O24.011, E10.69, E10.638, E09.40, E09.351, E08.622, E08.42, O24.913, O24.019, E11.00, E10.52, E09.630, E09.42, E09.36, E08.69, E08.610, E08.349, E08.29, O24.420, O24.013, E10.618, E09.69, E09.628, E09.10, E08.638, E08.11, O24.82, O24.33, O24.313, E13.649, E11.630, E11.52, E10.65, E09.9, E09.610, E09.43, E09.331, E08.620, E08.311, E08.21, O24.435, O24.425, O24.415, E11.37X9, E11.37X3, E11.37X2, E11.37X1, E11.37, E09.37X9, E09.37X3, E09.37X2, E09.37X1, E09.37, E09.3599, E09.3593, E09.3592, E09.3591, E09.3559, E09.3553, E09.3552, E09.3551, E09.355, E09.3549, E09.3543, E09.3542, E09.3541, E09.354, E09.3539, E09.3533, E09.3532, E09.3531, E09.353, E09.3529, E09.3523, E09.3522, E09.3521, E09.352, E09.3519, E09.3513, E09.3512, E09.3511, E09.3499, E09.3493, E09.3492, E09.3491, E09.3419, E09.3413, E09.3412, E09.3411, E09.3399, E09.3393, E09.3392, E09.3391, E09.3319, E09.3313, E09.3312, E09.3311, E09.3299, E09.3293, E09.3292, E09.3219, E09.3213, E09.3212, E09.3211, E08.37X9, E08.37X3, E08.37X2, E08.37X1, E08.37, E08.3599, E08.3593, E08.3592, E08.3591, E08.3559, E08.3553, E08.3552, E08.3551, E08.355, E08.3549, E08.3543, E08.3542, E08.3541, E08.354, E08.3539, E08.3533, E08.3532, E08.3531, E08.353, E08.3529, E08.3523, E08.3522, E08.3521, E08.352, E08.3519, E08.3513, E08.3512, E08.3511, E08.3499, E08.3493, E08.3492, E08.3491, E08.3419, E08.3413, E08.3412, E08.3411, E08.3393, E08.3392, E08.3391, E08.3313, E08.3312, E08.3311, E08.3299, E08.3293, E08.3292, E08.3291, E08.3219, E08.3213, E08.3212, E08.3211, P70.2, E13.9, E13.8, E11.9, E11.8, E10.9, E10.8, O24.91, O24.9, O24.81, O24.8, O24.43, O24.42, O24.41, O24.4, O24.31, O24.3, O24.11, O24.1, O24.01, O24.0, O24, E13.64, E13.63, E13.61, E13.6, E13.1, E13.0, E13, E11.64, E11.63, E11.61, E11.6, E11.0, E11, E10.64, E10.63, E10.61, E10.6, E10.1, E10, E09.64, E09.63, E09.62, E09.61, E09.6, E09.5, E09.4, E09.35, E09.34, E09.33, E09.32, E09.31, E09.3, E09.2, E09.1, E09.0, E09, E08.64, E08.63, E08.62, E08.61, E08.6, E08.5, E08.4, E08.35, E08.34, E08.32, E08.31, E08.3, E08.2, E08.1, E08.0, E08, E11.10, E11.1 | 648, 250.2, 250.02, 250, 249.51, 249.31, 249, 648.01, 249.71, 249.41, 249.3, 249.1, 249.1, 250, 249.4, 250, 249.4, 648.02, 648, 250.21, 249.3, 249.01, 249, 249.2, 648.04, 249.91, 249.6, 249.9, 249.9, 249.8, 249.7, 249.5, 249.81, 249.6, 250.22, 249.61, 249.5, 648.03, 250.03, 249.8, 249.7, 250.01, 249.11, 249, 775.1, 249.21, 249.2 | C10Q.00, C10M.00, C10F411, C10EH00, C10EE11, C109J11, C109G12, C100011, C10K000, C108512, C10P000, C10ER00, C10EE12, C10E611, C108E11, L180900, C10y100, C10H.00, C10FD11, C109F11, C109512, C100112, C100000, C10E912, C10E312, C10A100, C109G00, C10J000, C108E12, C1zy600, C10P111, C10P100, C10FF00, C10F500, C10A500, C11y000, C10yy00, C10EA12, C10E911, C10B000, C10AW00, C109G11, C109400, C108H12, C108.00, C10..00, L180800, L180300, C10P.00, C10F900, C10EA00, C109H00, C109F00, C108A12, C100111, L180z00, C10yz00, C10N000, C10M000, C10EE00, C109D12, C109911, C109.11, C109.00, C102000, L180X00, C10H000, C10E511, C109J00, C109411, L180400, Cyu2100, C10L.00, C10F.11, C10AX00, C10A600, C10A.11, C109500, PKyP.00, L180600, L180000, C1zy311, C10FG00, C10D.11, C109D11, C10E.00, C10A700, C10A200, C109J12, C108.11, C100100, L180700, C10FS00, C10D.00, C10C.12, C109H11, C108A00, Cyu2.00, C10J.00, C10FH11, C10FF11, C109D00, C109912, C109412, C108z00, C108E00, C108611, Q441.00, C10FG11, C10F911, C108A11, Cyu2000, C10y000, C108y00, C10G.00, C10E600, C108511, C102100, C100z00, L180100, C10E.11, C109511, C109.13, C108911, C10K.00, C10F400, C10G000, C10EH11, L180811, L180.00, C10FJ11, C10FJ00, C10C.00, C10B.00, C10A.00, L180200, C1zy300, C10P011, C10FD00, C10EA11, C10E900, C109F12, C108612, C108300, C108.13, PKyP.11, Lyu2900, C10N.00, C10EH12, C108H11, C108912, C108900, Cyu2200, C10E500, C10N100, C10E.12, C108H00, C100.00, C10F511, C10C.11, L180500, C10FH00, C10F.00, C109H12, C109900, C109.12, C108.12, L180B00, L180A00 |
| Type 1 Diabetes | 45766051, 45757674, 43531009, 43531008, 42535539, 37018566, 36717215, 4152858, 4151281, 4145827, 4102018, 4099215, 4099214, 4096668, 4063042, 4047906, 443412, 201531, 201254 | E10.621, E10.649, E10.10, O24.012, E10.622, O24.03, E10.610, E10.630, O24.011, E10.69, E10.638, O24.019, E10.52, O24.013, E10.618, E10.65, E10.9, E10.8, O24.01, O24.0, E10.64, E10.63, E10.61, E10.6, E10.1, E10 | 250.01, 250.03, 250.21 | C10EH00, C10EE11, C100011, C108512, C10P000, C10ER00, C10EE12, C10E611, C108E11, C100000, C108E12, C10EA12, C10E911, C108H12, C108.00, C10EA00, C108A12, C10EE00, C102000, C10E511, C10E.00, C108.11, C108A00, C108E00, C108611, C108A11, C10y000, C10E600, C108511, C10E.11, C108911, C10EH11, C10P011, C10EA11, C10E900, C108612, C108.13, C10EH12, C108H11, C108912, C10E500, C10E.12, C108H00, L180500, C108.12 |
| Type 2 Diabetes | 45766052, 45757474, 43531010, 36714116, 4321756, 4304377, 4230254, 4200875, 4198296, 4196141, 4193704, 4130162, 4129519, 4099651, 4099217, 4063043, 201826, 201530 | O24.119, E11.65, E11.622, E11.610, E11.69, E11.22, E11.649, O24.111, E11.618, E11.01, O24.13, O24.112, E11.621, O24.113, E11.638, E11.641, E11.00, E11.630, E11.52, E11.37X9, E11.37X3, E11.37X2, E11.37X1, E11.37, E11.9, E11.8, O24.11, O24.1, E11.64, E11.63, E11.61, E11.6, E11.0, E11, E11.10, E11.1 | 250.00, 250.02, 250.20, 250.22 | C10F411, C109J11, C109G12, C10FD11, C109F11, C109512, C100112, C109G00, C10P111, C10P100, C10FF00, C10F500, C109G11, C109400, C10F900, C109H00, C109F00, C100111, C109D12, C109911, C109.11, C109.00, C109J00, C109411, C10F.11, C109500, L180600, C10FG00, C109D11, C109J12, C100100, C109H11, C10FH11, C10FF11, C109D00, C109912, C109412, C10FG11, C10F911, C102100, C109511, C109.13, C10F400, C10FJ11, C10FJ00, C10FD00, C109F12, C10F511, C10FH00, C10F.00, C109H12, C109900, C109.12, L180B00 |
| Hypertension [Part 1 of treated hypertension – must have hypertension codes in prior year and part 2 (antihypertensive) in prior 30 days] | 45757787, 4321603, 4302591, 4269358, 4263067, 4217486, 4215640, 4180283, 4159755, 4148205, 4083723, 4058987, 4034031, 320128, 317898, 312648 | I10 | 401, 401.0, 401.1, 401.9 | G20..00, G20..12, G200.00, G201.00, G20z.00 |
| Proteinuria | 42689513, 37016364, 36712846, 36712845, 4338683, 4298811, 4265332, 4262273, 4252269, 4252096, 4247664, 4237908, 4231294, 4228055, 4211056, 4207847, 4205617, 4205574, 4198209, 4195061, 4185287, 4180750, 4173241, 4168230, 4156996, 4147279, 4140559, 4137160, 4132125, 4131811, 4128528, 4119281, 4099759, 4075748, 4059585, 4059124, 4059122, 4059121, 4056483, 4056482, 4056481, 4055902, 4055901, 4033489, 4008125, 75650, 74080 | R80.9, R80.8, R80.1, R80.3, R80.0, R80.2, N06.9, N06.8, N06.7, N06.6, N06.5, N06.4, N06.3, N06.2, N06.1, N06.0, R80, N06 | 791.0, 593.6 | R110z00, K0A4000, K0A4500, K0A4300, K0A4100, K0A4700, K0A4400, K0A4.00, K0A4600, K190X00, R110200, Kyu5G00, K136.00, K0A4200, K136.11, 4678.00, R110300, R110.00, R110100 |
| eGFR<45 or end stage renal disease | 46273164, 45772751, 45769906, 45769904, 45768813, 45757393, 45757392, 44782717, 43021864, 43020455, 37018886, 4128200, 4125970, 4030520, 762973, 193782 | N18.6 | 585.6, 403.01 | K05..12, K050.00, K0D..00 |
| Former smoker | 37109024, 37109023, 4103418, 4103417, 4099811, 765685, 765451, 764471, 764470, 764469, 764468, 437264 | O99.335, F17.221, F17.201, O99.330, F17.211, F17.291, O99.33 | 305.1 | E251.00, E251000, E251100, E251200, E251300, E251z00 |
| Family history of coronary heart disease | 44789401 |  | - | - |
| Atrial fibrillation | 45768480, 44782442, 37395821, 4232697, 4232691, 4199501, 4154290, 4141360, 4119602, 4119601, 4117112, 4108832, 313217 | I48.91, I48.1, I48.0, I48.9, I48.2, I48, I48.21, I48.20, I48.19, I48.11 | 427.3, 427.31 | G573300, G573200, G573000, G573z00, G573500, G573.00, G573400, G573700 |
| Coronary heart disease | 4113303, 4109649, 4109491, 4109490, 4109489, 4208928, 4265210, 43022038, 4020601, 44783200, 37115756, 4121641, 45766238, 44784623, 36712779, 4252385, 4048534, 4046022, 764507, 316995, 4304192, 4225958, 4209308, 4153091, 4134723, 4108215, 761921, 4127089, 315830, 42536628, 4124846, 45766267, 43021619, 43021618, 43021617, 43021616, 43020480, 40491974, 40491973, 4188546, 4187852, 4160783, 4160782, 4160781, 4113302, 4109648, 4109488, 4109487, 4108875, 4030427, 4029827, 4029362, 4029361, 4027245, 4016142, 4270354, 46269996, 44806109, 42872402, 40481919, 37016181, 36714444, 36712983, 36712982, 4242670, 4199962, 4178622, 4178321, 4175846, 4168972, 4161455, 4155962, 4155007, 4124682, 4119951, 4111393, 4108673, 764149, 764123, 317576, 43022037, 43022026, 43022025, 43021973, 43021937, 43021888, 43021886, 43021885, 43021796, 43021795, 43021631, 43021630, 43021628, 43021627, 43021615, 43021582, 43021581, 43021580, 43021579, 43021578, 43021574, 43021572, 43021570, 43021476, 43021475, 43021474, 43021472, 43021470, 43021437, 43021436, 43021435, 43021434, 43021432, 43021314, 43021313, 43021312, 43021311, 43021310, 43021309, 43021308, 43021307, 43021306, 43021305, 43021304, 43021303, 43021302, 43021301, 43021294, 43021193, 43020880, 43020689, 43020580, 43020579, 43020576, 43020575, 43020574, 43020573, 43020572, 43020571, 43020570, 43020569, 43020561, 42873052, 42873051, 42873050, 40491973, 4329430, 4328721, 4253217, 4206334, 4178750, 4152843, 4113303, 4113301, 4113300, 4113299, 4113298, 4113297, 4109649, 4109647, 4109646, 4109644, 4109642, 4109491, 4109487, 4109486, 4109485, 4109484, 4109483, 4108876, 4108874, 4108873, 4108872, 4069186, 4069185, 4030427, 4029827, 4029362, 4029361, 4027245, 4016142, 321109, 42536629, 42536628, 4069185, 316427, 43022045, 4243371, 4119613, 4108172, 4097848, 4069186 | I25.791, I25.110, I25.82, I25.721, I25.119, I25.751, I25.711, I25.111, I25.10, I25.118, I25.84, I25.41, I25.83, I25.701, I25.761, I25.731, I25.42, Q24.5, I24.0, I20.1, I25.81, I25.4, I25.11, I25.1 | 414.2, 411.81, 414.0, 414.00, 414.01, 414.3, 414.4, 746.85, 414.11 | P6y4600, G341200, P6y4500, 6y4300, P6y4100, P6y4z00, P6y4400, P6y4411, P6y4000, P6y4200, P6y4.00, P6y4500, P6y4600, G340000, G340.12, G340.00, G3...12, G340.11, G39..00, G3...11, G340100, G34z000, G704.00, P6y4411, G331.11, G331.00, G332.00, G311011, G30..12, G311000, G312.00, G30..16, SP07600, G72B100, SP00300 |
| Congestive cardiac failure | 44784442, 44784345, 44782728, 44782713, 44782655, 44782428, 43022068, 43021826, 43021825, 36713488, 36712928, 36712927, 4327205, 4284562, 4242669, 4229440, 4215446, 4206009, 4184497, 4142561, 4139864, 4023479, 764874, 762003, 762002, 439698, 439696, 439694, 319835, 316994, 315295, 314378 | I50.30, I50.41, I50.42, I50.20, I50.22, I50.43, I50.23, I50.33, I50.31, I50.21, I50.40, I50.32, I13.0, I50.4, I50.3, I50.2, I50.82 | 402.01, 402.91, 428.0, 404.93, 402.11, 398.91, 404.13, 404.91, 404.11, 404.03, 404.01 | G580100, G580.11, G580.00, G211100, G1yz100, G580400, G210100, G234.00, G580000, G232.00, G580.14 |
| Rheumatoid arthritis | 42539550, 42536657, 42534837, 42534836, 42534835, 42534834, 37395590, 37117421, 37108714, 37108591, 37108590, 4347065, 4344494, 4343936, 4311391, 4297651, 4296152, 4271003, 4269880, 4200987, 4179536, 4179378, 4162539, 4147418, 4117687, 4117686, 4116446, 4116445, 4116444, 4116443, 4116442, 4116441, 4116440, 4116439, 4116153, 4116152, 4116151, 4116150, 4116149, 4116148, 4115161, 4115051, 4115050, 4114444, 4114442, 4114441, 4114440, 4114439, 4103516, 4083556, 4035611, 4035427, 81097, 80809, 78230 | M08.972, M08.931, M08.912, M08.872, M08.842, M08.841, M08.449, M08.419, M08.242, M08.222, M08.079, M08.071, M08.061, M08.052, M08.041, M06.871, M06.841, M06.259, M06.242, M06.239, M06.20, M06.08, M06.041, M06.031, M06.011, M05.851, M05.741, M05.731, M05.672, M05.669, M05.661, M05.512, M05.449, M05.422, M05.242, M05.239, M05.219, M05.179, M05.011, M08.939, M08.469, M08.439, M08.421, M08.29, M08.232, M08.229, M08.049, M08.039, M08.031, M06.861, M06.819, M06.252, M06.222, M06.061, M05.852, M05.849, M05.822, M05.821, M05.679, M05.632, M05.621, M05.349, M05.211, M05.171, M08.269, M08.211, M06.829, M06.211, M06.00, M05.832, M05.779, M05.771, M05.742, M05.711, M05.529, M05.519, M05.442, M05.419, M05.412, M05.411, M05.29, M05.259, M05.241, M05.221, M08.839, M08.821, M08.471, M08.452, M08.221, M08.212, M06.872, M06.212, M06.019, M05.772, M05.662, M05.641, M05.629, M05.622, M05.551, M05.541, M05.532, M05.469, M05.271, M05.269, M05.229, M05.141, M05.112, M05.10, M05.051, M08.949, M08.851, M08.462, M08.279, M08.272, M08.252, M08.029, M06.89, M06.272, M06.261, M06.251, M05.872, M05.841, M05.372, M05.30, M05.222, M05.111, M05.00, M08.239, M05.842, M05.749, M05.732, M05.572, M05.562, M05.559, M05.552, M05.432, M05.341, M05.339, M05.172, M05.132, M05.122, M05.119, M05.031, M08.879, M08.812, M08.461, M08.451, M08.262, M08.241, M08.231, M06.849, M06.839, M06.831, M06.271, M06.249, M06.221, M06.219, M06.071, M06.059, M06.049, M06.039, M06.029, M06.022, M05.631, M05.49, M05.439, M05.39, M05.251, M05.161, M05.149, M05.142, M05.09, M05.069, M05.052, M08.459, M08.062, M08.022, M06.811, M06.29, M06.232, M06.09, M06.042, M05.831, M05.751, M05.722, M05.649, M05.642, M05.479, M05.452, M05.371, M05.361, M05.332, M05.261, M05.20, M05.062, M08.942, M08.479, M08.251, M08.072, M08.012, M06.88, M06.879, M06.859, M06.832, M06.812, M06.072, M05.759, M05.719, M05.69, M05.659, M05.59, M05.521, M05.461, M05.431, M05.369, M05.312, M05.262, M05.169, M05.139, M05.049, M05.032, M05.019, M08.961, M08.929, M08.911, M08.871, M08.861, M08.822, M08.431, M08.042, M06.862, M06.842, M06.822, M06.279, M06.269, M06.262, M06.032, M05.811, M05.729, M05.569, M05.421, M05.362, M05.342, M05.331, M05.311, M05.232, M05.121, M05.022, M08.952, M08.922, M08.921, M08.869, M08.862, M08.849, M08.811, M08.442, M08.441, M08.429, M08.411, M08.069, M06.231, M06.051, M06.021, M05.819, M05.639, M05.471, M05.429, M05.379, M05.352, M05.319, M05.19, M05.021, M08.979, M08.962, M08.941, M08.919, M08.831, M08.819, M08.019, M06.062, M05.89, M05.862, M05.839, M05.80, M05.79, M05.739, M05.671, M05.619, M05.579, M05.322, M05.321, M05.212, M05.162, M05.079, M08.932, M08.832, M08.829, M08.432, M08.422, M08.412, M08.271, M08.261, M08.249, M08.219, M08.09, M06.869, M06.80, M05.879, M05.871, M05.869, M05.762, M05.761, M05.721, M05.651, M05.60, M05.561, M05.531, M05.459, M05.359, M05.152, M05.151, M05.072, M05.061, M05.042, M05.041, M05.029, M08.971, M08.852, M08.032, M08.021, M06.852, M06.851, M06.241, M05.861, M05.712, M05.70, M05.549, M05.451, M05.231, M05.131, M05.129, M05.059, M08.859, M08.059, M08.051, M06.821, M06.229, M06.069, M05.752, M05.571, M05.542, M05.539, M05.511, M05.472, M05.351, M05.279, M05.272, M05.249, M05.159, M05.039, M08.969, M08.959, M08.951, M08.472, M08.259, M06.079, M06.052, M06.012, M05.859, M05.829, M05.812, M05.769, M05.652, M05.522, M05.462, M05.441, M05.329, M05.252, M05.071, M05.012, M06.9, M05.9, M08.94, M08.93, M08.41, M08.26, M08.25, M08.23, M08.22, M08.06, M08.03, M08.02, M06.87, M06.86, M06.85, M06.84, M06.83, M06.82, M06.81, M06.8, M06.27, M06.26, M06.25, M06.24, M06.23, M06.22, M06.21, M06.07, M06.06, M06.05, M06.04, M06.03, M06.02, M06.01, M06.0, M06, M05.87, M05.86, M05.85, M05.84, M05.83, M05.82, M05.81, M05.8, M05.77, M05.76, M05.75, M05.74, M05.73, M05.72, M05.71, M05.7, M05.67, M05.66, M05.65, M05.64, M05.63, M05.62, M05.61, M05.6, M05.57, M05.56, M05.55, M05.54, M05.53, M05.52, M05.51, M05.47, M05.46, M05.45, M05.44, M05.43, M05.42, M05.41, M05.37, M05.36, M05.35, M05.34, M05.33, M05.32, M05.31, M05.3, M05.27, M05.26, M05.25, M05.24, M05.23, M05.22, M05.21, M05.2, M05.17, M05.16, M05.15, M05.14, M05.13, M05.12, M05.11, M05.1, M05.07, M05.06, M05.05, M05.04, M05.03, M05.02, M05.01, M05.0, M05 | 714.0, 714.1, 714.2 | N040J00, N047.00, N040P00, N041.00, N040E00, N040500, N040A00, N040900, N040800, N040C00, N04y011, N040L00, Nyu1G00, N040H00, N040B00, N040200, G5yA.00, N040K00, N040F00, N040700, N040G00, N040.00, Nyu1000, N040D00, N040000, N040600, N040T00, Nyu1200, N040N00, Nyu1100, N042z00, N040S00, N040400, N040300, N04..00, N04X.00, N042.00, N040M00 |
| Chronic renal disease | 6287169, 46286992, 46284603, 46284602, 46284600, 46284599, 46284598, 46284597, 46284593, 46284592, 46284591, 46284588, 46284587, 46284575, 46284572, 46284570, 46284567, 46284566, 46273636, 46273514, 46271022, 46270356, 46270355, 46270354, 46270353, 45773688, 45773576, 45771075, 45771067, 45771064, 45769903, 45769902, 45769901, 45763855, 45763854, 45757447, 45757446, 45757445, 45757444, 45757356, 45757139, 45757137, 44792255, 44792254, 44792253, 44792252, 44792251, 44792250, 44792249, 44792232, 44792231, 44792230, 44792229, 44792228, 44792227, 44792226, 44784640, 44784639, 44784638, 44784637, 44784621, 44782703, 44782692, 44782691, 44782690, 44782689, 44782429, 43531653, 43531578, 43531577, 43531566, 43531562, 43531559, 43021854, 43021853, 43021852, 43021836, 43021835, 43020457, 43020456, 43020437, 42872405, 37019193, 37018761, 37017813, 37017104, 36717534, 36717349, 36716947, 36716455, 36716184, 4322556, 4239804, 4128067, 765536, 765535, 764011, 762034, 762033, 762001, 762000, 760850, 443961, 443614, 443612, 443611, 443601, 443597, 198185 | I13.11, E11.22, O10.311, O10.213, O10.33, O10.312, O10.211, O10.32, O10.313, E08.22, O10.212, I13.10, O10.319, O10.23, O10.22, O10.219, N18.9, N18.5, N18.4, N18.3, N18.2, N18.1, I13.2, I12.9, O10.31, O10.21, O10.2, N18, I13.1, I13, I12 | 585.5, 585.1, 585.9, 404.9, 404, 403, 285.21, 585.2, 585, 403.91, 585.3, 404, 585.4, 404.1 | 1Z1T.00, 1Z1Q.00, 1Z1V.00, 1Z1X.00, 1Z1Y.00, 1Z1b.00, 1Z1M.00, 1Z1c.00, 1Z1R.00, 1Z1N.00, 1Z1S.00, 1Z1f.00, 1Z1a.00, 1Z1Z.00, 1Z1e.00, 1Z1W.00, 1Z1P.00, 1Z1d.00, 1Z1A.00, K051.00, 1Z1F.00, 1Z1A.11, 1Z17.00, 1Z11.00, K0E..00, 1Z1K.00, 1Z1E.00, K05..11, 1Z1D.00, 1Z16.00, 1Z1L.11, 1Z1B.00, 1Z1J.00, 1Z1H.00, 1Z15.00, D215000, 1Z1J.11, K053.00, K05..13, 1Z1E.11, 1Z1L.00, Kyu2100, 1Z1C.11, 1Z1C.00, 1Z19.11, 1Z19.00, 1Z18.00, 1Z1G.00, 1Z14.00, 1Z13.00, K052.00, 1Z17.11, K055.00, K054.00, 1Z1F.11, 1Z1..00, K05..00, 1Z1B.11, 1Z10.00, 1Z12.00, 1Z1K.11, 1Z18.11, 1Z1H.11, 1Z1G.11, 1Z1D.11 |
| Valvular heart disease | 45773168, 45773080, 45773076, 45771327, 45771326, 45771323, 45771320, 45771318, 45771317, 45769461, 45766240, 45766214, 45766213, 45766212, 45766211, 45766210, 45766209, 45766208, 45766207, 45766206, 45766205, 45766186, 45766185, 45766182, 45766181, 45766180, 45766179, 45766178, 45766177, 45766153, 45766136, 45766127, 45766126, 45766125, 45766124, 45766103, 45766100, 45766099, 45766097, 45766096, 45766095, 45766094, 45766093, 45766092, 45766089, 45766087, 45766086, 45766084, 45766083, 45766082, 45766081, 45766080, 45766079, 45766078, 45766072, 45766071, 45766069, 45757194, 45757096, 44813665, 44804855, 44804854, 44804853, 44804852, 44804851, 44804848, 44804847, 44804762, 44804761, 44804760, 44804759, 44804758, 44804757, 44804756, 44804755, 44804754, 44804753, 44804752, 44804751, 44804750, 44804745, 44803307, 43022061, 43022049, 43022048, 43022047, 43022027, 43021953, 43021947, 43021946, 43021945, 43021944, 43021943, 43021941, 43021898, 43021897, 43021896, 43021895, 43021894, 43021893, 43021892, 43021891, 43021890, 43021833, 43021806, 43021803, 43021713, 43021711, 43021710, 43021709, 43021708, 43021625, 43021624, 43021479, 43021473, 43021336, 43021327, 43021326, 43021325, 43021324, 43021071, 43021070, 43021063, 43021061, 43021051, 43021049, 43021048, 43021045, 43021044, 43021043, 43020964, 43020963, 43020962, 43020961, 43020960, 43020959, 43020958, 43020957, 43020956, 43020955, 43020951, 43020950, 43020949, 43020948, 43020947, 43020946, 43020945, 43020923, 43020882, 43020881, 43020688, 43020641, 43020640, 43020639, 43020638, 43020637, 43020636, 43020635, 43020634, 43020633, 43020632, 43020631, 43020630, 43020629, 43020628, 43020627, 43020626, 43020625, 43020624, 43020623, 43020622, 43020621, 43020620, 43020619, 43020618, 43020617, 43020616, 43020504, 43020466, 43020465, 43020459, 42539373, 42539157, 42538803, 42538759, 42538671, 42538531, 42537638, 42535092, 40493497, 40493487, 40493477, 40493476, 40493473, 40493470, 40493460, 40493459, 40493454, 40493451, 40493447, 40493446, 40493444, 40493431, 40493430, 40493418, 40493417, 40492991, 40492988, 40492983, 40492982, 40492979, 40492960, 40492956, 40492953, 40492502, 40492501, 40492500, 40492470, 40492455, 40492453, 40492189, 40491995, 40491986, 40491977, 40491976, 40491971, 40491970, 40491968, 40491967, 40491938, 40491868, 40491529, 40491519, 40491515, 40491481, 40491480, 40491479, 40491478, 40491457, 40491456, 40491011, 40490966, 40490956, 40490947, 40490938, 40490515, 40490483, 40490430, 40489995, 40489971, 40489903, 40489423, 40489419, 40489373, 40489368, 40488970, 40488873, 40488841, 40488801, 40488797, 40488795, 40488469, 40488468, 40488467, 40488444, 40488437, 40488397, 40488396, 40488395, 40488394, 40488079, 40488075, 40488037, 40488036, 40488033, 40488032, 40488026, 40488014, 40488012, 40488008, 40488007, 40488006, 40487992, 40487980, 40487979, 40487966, 40487672, 40487660, 40487654, 40487653, 40487652, 40487649, 40487643, 40487625, 40487624, 40487600, 40487571, 40487547, 40487186, 40487184, 40487183, 40487182, 40487107, 40487083, 40487038, 40486743, 40486738, 40486737, 40486733, 40486706, 40486664, 40486663, 40486635, 40486626, 40486625, 40486616, 40486615, 40486608, 40486559, 40486554, 40486225, 40486179, 40486135, 40486132, 40486073, 40486072, 40486071, 40486070, 40486057, 40486056, 40486055, 40486045, 40486044, 40486042, 40484305, 40484273, 40483337, 40483294, 40483289, 40482926, 40482925, 40482912, 40482911, 40482910, 40482894, 40482888, 40481625, 40481579, 40404007, 37397481, 37396391, 37111410, 37109669, 37109597, 36716985, 36716030, 36715308, 36715123, 36714644, 4354071, 4354070, 4354066, 4354065, 4354064, 4354063, 4353836, 4353822, 4353743, 4353740, 4339544, 4334321, 4332658, 4331802, 4331669, 4329095, 4329088, 4326746, 4324704, 4324608, 4322316, 4315010, 4314505, 4313828, 4312621, 4305961, 4304541, 4301771, 4301373, 4300644, 4300540, 4298429, 4294530, 4290023, 4289152, 4281749, 4269199, 4263915, 4263730, 4263510, 4262976, 4261611, 4259295, 4252872, 4246514, 4245933, 4245920, 4245811, 4245810, 4245803, 4245692, 4245443, 4245321, 4245227, 4245104, 4244966, 4244935, 4244437, 4244374, 4244184, 4244173, 4244027, 4242188, 4242177, 4242036, 4240733, 4240289, 4239569, 4238986, 4238914, 4237202, 4232337, 4231819, 4231818, 4231452, 4230774, 4227091, 4226024, 4226023, 4225090, 4222174, 4221806, 4220765, 4219047, 4218450, 4218343, 4215741, 4215619, 4213379, 4209011, 4203920, 4197259, 4195690, 4195677, 4195003, 4189522, 4189343, 4188554, 4187858, 4184499, 4184129, 4183844, 4182602, 4182346, 4182110, 4181949, 4179187, 4178585, 4178551, 4178128, 4177860, 4176161, 4176099, 4175807, 4175580, 4172866, 4171708, 4171268, 4169568, 4164798, 4163292, 4161975, 4161459, 4161458, 4161245, 4160899, 4158911, 4156119, 4155964, 4154293, 4152613, 4152384, 4148905, 4147787, 4146080, 4144901, 4143971, 4143921, 4143607, 4141491, 4133509, 4125008, 4125007, 4125006, 4125005, 4125004, 4124690, 4123286, 4122764, 4122085, 4121472, 4121471, 4121469, 4120279, 4120278, 4120277, 4119957, 4119956, 4119955, 4119589, 4119463, 4119462, 4119461, 4119459, 4117862, 4117268, 4117132, 4112096, 4112093, 4112092, 4112091, 4112090, 4112089, 4112088, 4112087, 4112086, 4112085, 4112084, 4112083, 4112017, 4112016, 4111566, 4111565, 4111416, 4111414, 4111413, 4111412, 4111409, 4111099, 4111098, 4110937, 4109466, 4109465, 4109463, 4109347, 4109341, 4109340, 4109339, 4109338, 4109337, 4109336, 4109335, 4109334, 4109202, 4109201, 4109200, 4109199, 4108819, 4108817, 4108815, 4108736, 4108732, 4108731, 4108730, 4108729, 4108728, 4108727, 4108726, 4108725, 4108724, 4108723, 4108659, 4108608, 4108603, 4108602, 4108601, 4108598, 4108597, 4108596, 4108595, 4108235, 4108234, 4108233, 4108220, 4108219, 4108170, 4108168, 4108166, 4108165, 4108164, 4108085, 4104799, 4103319, 4103187, 4103131, 4103129, 4103015, 4103014, 4103013, 4103012, 4103010, 4103009, 4103008, 4103006, 4103005, 4103004, 4103003, 4103002, 4103000, 4102999, 4102998, 4102997, 4102996, 4102995, 4102994, 4102993, 4102992, 4102991, 4102989, 4102988, 4102867, 4102866, 4102865, 4102864, 4102863, 4102862, 4102861, 4102860, 4102859, 4102857, 4101633, 4101620, 4101619, 4101618, 4101616, 4101615, 4101614, 4101613, 4101612, 4101611, 4101609, 4101608, 4101607, 4101606, 4101605, 4101494, 4101493, 4101492, 4101491, 4101490, 4101489, 4101488, 4101486, 4101485, 4101484, 4101483, 4101482, 4101481, 4101315, 4101314, 4101313, 4101189, 4101188, 4101187, 4101186, 4101185, 4101184, 4101183, 4101182, 4101181, 4101179, 4101178, 4101177, 4101176, 4101175, 4101174, 4101173, 4101171, 4101170, 4101169, 4101168, 4101167, 4101166, 4101165, 4101164, 4101162, 4101161, 4101160, 4101036, 4101035, 4101034, 4101013, 4100882, 4100880, 4100879, 4100878, 4100877, 4100876, 4100875, 4100277, 4100276, 4100275, 4100274, 4100273, 4100272, 4100271, 4100270, 4100269, 4100268, 4100267, 4100266, 4100265, 4100264, 4100263, 4100262, 4100261, 4100260, 4100259, 4100258, 4100256, 4100255, 4100153, 4100152, 4100151, 4100150, 4100149, 4100148, 4100146, 4100145, 4100144, 4100143, 4097813, 4081037, 4078214, 4070926, 4070916, 4070914, 4070821, 4070798, 4070795, 4070297, 4069182, 4069180, 4069096, 4066746, 4058643, 4058384, 4057759, 4049296, 4048535, 4048213, 4048042, 4046114, 4046113, 4040820, 4035467, 4033905, 4030224, 4020602, 4020159, 4012483, 4011131, 4006971, 762783, 762045, 762035, 443962, 439834, 438777, 438171, 437897, 437461, 435829, 433010, 432937, 321107, 321041, 320835, 320429, 320416, 320206, 320115, 319921, 319845, 319843, 318776, 318689, 317890, 317296, 316993, 315564, 315282, 315273, 314457, 314368, 314054, 313964, 313698, 313416, 313221, 313007, 313006, 313005, 312728 | T82.09XD, T82.09XA, T82.03XD, T82.02XD, T82.6XXA, T82.02XA, T82.01XA, T82.03XA, T82.6XXD, T82.01XD, Q25.3, Q23.3, Q23.2, Q23.1, Q23.0, Q22.9, Q22.8, Q22.5, Q22.4, Q22.3, Q22.2, Q22.1, Q22.0, Q21.3, Q21.2, I51.2, I51.1, I38, I37.9, I37.8, I37.2, I37.1, I37.0, I36.9, I36.8, I36.2, I36.1, I36.0, I35.9, I35.8, I35.2, I35.1, I35.0, I34.9, I34.8, I34.2, I34.1, I34.0, I23.5, I23.4, I09.1, I08.9, I08.8, I08.3, I08.2, I08.1, I08.0, I07.9, I07.8, I07.2, I07.1, I07.0, I06.9, I06.8, I06.2, I06.1, I06.0, I05.9, I05.8, I05.2, I05.1, I05.0, I01.1, T82.0, Q22, I37, I36, I35, I34, I08, I07, I06, I05, T82.6, T82.09, T82.03, T82.02, T82.01 | 394.1, 391.1, 93.23, 424.3, 746, 424.2, 746.02, 424.99, 397, 395.2, 429.81, 424, 396, 394, 93.21, 746.01, 395, 394.2, 394, 996.71, 746.3, 394.9, 746.6, 746.2, 424.9, 396.8, 396.1, 396.2, 746.4, 396.3, 93.22, 429.5, 746.09, 424.9, 93.24, 429.6, 395.1, 746.1, 745.2, 424.1, 397.9, 397.1, 395.9, 395, 746.5, 746, 396.9, 396 | 62..00, P60zz00, P602000, P52..00, Gyu5C00, G54z500, G544200, G544100, G544.00, G543.00, G140200, G122.00, G540.15, G121.12, Pyu2200, P65z.00, P60z.00, G543200, G140511, G12..00, G112.00, P61..00, Gyu5700, G54z014, G543000, G541z00, G541700, G120.00, G11..11, P6z0.00, Gyu5A00, G540.12, G121.00, P60z000, G541100, G540100, G111.11, P640.00, G543z00, G542.00, G541600, G140413, G11z.00, G110.00, A932400, P61z.00, Gyu1100, G544X00, G543215, G141100, Pyu2G00, P6X..00, G543311, G540.16, G14z.00, G133.00, G131.13, P6yyC00, Gyu1200, G54z200, G54z013, G54z000, G140412, G140400, P60..00, P553.00, G140000, Pyu2400, P722411, P60z200, P52z.00, Gyu1500, G5y5.00, G541400, G541211, G541.00, SP00400, Pyu2300, P722400, P6W..00, P653.00, P60z100, G71A.00, G5yy100, G5y6.00, G540200, G140100, P520.12, G54z100, G541200, G541011, G140z00, P6yy.11, P602.00, Gyu1000, G543300, G542000, G54..11, G141.00, G132.12, P65..11, P65..00, P520.00, Gyu5D00, G542200, G11..00, G54z.00, G141z00, G13y.00, G110.11, P610.00, P601z00, P601000, P521.00, Gyu5500, G541500, G540300, G130.00, G121.11, Pyu2H00, G54zz00, G54z300, G543011, G140300, G14..00, G111.12, A932100, SP00200, P64z.00, Gyu5f00, Gyu5900, G14021Y, G140111, G133.11, P72z000, P63..00, G544000, G542100, G541300, G540.14, G13z.00, G112.13, P66..00, P602z00, G5yy300, G540000, G112.12, Gyu5B00, G113.00, G111.00, G011.00, A932200, P602100, P600.00, G5yy000, G543213, G542z00, G541012, G140500, G131.00, P641.00, Gyu5800, G140112, G131.14, P64..00, G543012, G542011, G541212, G30y100, G140514, G12z.00, A932300, P650.00, G5yy400, G5yy200, G365.00, G364.00, G133.12, G132.13, G132.00, G13..00, G543100, G540z00, G141200, P611.00, P520.11, Gyu5600, G5yy.11, G542X00, G542012, G541000, G540.00, A932.11, P652.00, Gyu1300, G140.00, P651.00, P601.00, G719.00, G543400, G141000, G14021X, G114.00 |
|  | **rxNorm ingredients** |  |  |  |
| Part 2 of treated hypertension – must have these codes in prior 30 days and part 1 (hypertension) in prior year | 44507580, 44506752, 19115050, 19101849, 19093166, 19085688, 19078808, 19062195, 19055531, 19027413, 19026448, 19022533, 19020994, 19011951, 19011021, 19005658, 1401581, 1398937, 1394333, 1387219, 1373928, 1363053, 1362225, 1350489, 1344965, 1337068, 1326378, 1321636, 1309068, 1305496, 1305447 |  |  |  |
