# Supplementary material for: Feasibility and evaluation of a large-scale external validation approach for patient-level prediction in an international data network: validation of models predicting stroke in female patients newly diagnosed with atrial fibrillation
Source: BMC Med Res Methodol. 2020 May 6;20:102. doi: 10.1186/s12874-020-00991-3 (PMC7201646; doi:10.1186/s12874-020-00991-3)
Supplement: Supplementary file 2 — Additional file 2. Appendix B. Snomed codes defining atrial fibrillation. [file 12874_2020_991_MOESM2_ESM.docx]

**APPENDIX B**

Snomed codes defining atrial fibrillation

| Id | Code | Name |
| --- | --- | --- |
| 45768480 | 706923002 | Longstanding persistent atrial fibrillation |
| 44782442 | 1.2004E+14 | Atrial fibrillation with rapid ventricular response |
| 37395821 | 715395008 | Familial atrial fibrillation |
| 4232697 | 440059007 | Persistent atrial fibrillation |
| 4232691 | 440028005 | Permanent atrial fibrillation |
| 4199501 | 314208002 | Rapid atrial fibrillation |
| 4154290 | 282825002 | Paroxysmal atrial fibrillation |
| 4141360 | 426749004 | Chronic atrial fibrillation |
| 4119602 | 233911009 | Non-rheumatic atrial fibrillation |
| 4119601 | 233910005 | Lone atrial fibrillation |
| 4117112 | 300996004 | Controlled atrial fibrillation |
| 4108832 | 195080001 | Atrial fibrillation and flutter |
| 313217 | 49436004 | Atrial fibrillation |

Codes defining ECG

| Id | Code | Name |
| --- | --- | --- |
| 46273550 | 709187004 | Electrocardiography via fetal scalp electrodes |
| 46271135 | 709186008 | Noninvasive abdominal fetal electrocardiography |
| 45772774 | 705012007 | Anterior and lateral ST segment elevation |
| 45767628 | 705096009 | Deep symmetric anterior T wave inversion |
| 45767627 | 705095008 | Inferior and right sided ST segment elevation |
| 45767560 | 705013002 | Inferior and lateral ST segment elevation |
| 45767559 | 705011000 | Anterior ST segment depression |
| 45767558 | 705010004 | Posterior ST segment elevation |
| 45767557 | 705009009 | Posterior ST segment depression |
| 45767556 | 705008001 | Anterior ST segment elevation |
| 45767555 | 705007006 | Inferior ST segment elevation |
| 45767554 | 705006002 | Lateral ST segment elevation |
| 45767549 | 704999008 | Anterior T wave inversion |
| 45767548 | 704998000 | Diffuse ST segment depression |
| 45767547 | 704997005 | Inferior ST segment depression |
| 45767546 | 704996001 | Inverted biphasic anterior T wave |
| 44808009 | 8.4741E+14 | Electrocardiogram rhythm strip monitoring |
| 44514180 | U30.1 | Tilt table testing |
| 44514102 | U19.9 | Unspecified diagnostic electrocardiography |
| 44514101 | U19.8 | Other specified diagnostic electrocardiography |
| 44514099 | U19.6 | Cardiomemo electrocardiographic monitoring |
| 44514098 | U19.5 | Holter extended electrocardiographic recording |
| 44514096 | U19.3 | 48 hour ambulatory electrocardiography |
| 44514095 | U19.2 | 24 hour ambulatory electrocardiography |
| 44513816 | R43.8 | Other specified ultrasound monitoring |
| 43527887 | 0298T | External electrocardiographic recording for more than 48 hours up to 21 days by continuous rhythm recording and storage; review and interpretation |
| 43527886 | 0297T | External electrocardiographic recording for more than 48 hours up to 21 days by continuous rhythm recording and storage; scanning analysis with report |
| 43527885 | 0296T | External electrocardiographic recording for more than 48 hours up to 21 days by continuous rhythm recording and storage; recording (includes connection and initial recording) |
| 43527884 | 0295T | External electrocardiographic recording for more than 48 hours up to 21 days by continuous rhythm recording and storage; includes recording, scanning analysis with report, review and interpretation |
| 40756895 | 0206T | Computerized database analysis of multiple cycles of digitized cardiac electrical data from two or more ECG leads, including transmission to a remote center, application of multiple nonlinear mathematical transformations, with coronary artery obstruction |
| 40491313 | 447114004 | 12 lead electrocardiogram during exercise |
| 40491312 | 447113005 | 12 lead electrocardiogram at rest |
| 40483798 | 445481009 | Bifascicular block on electrocardiogram |
| 40483359 | 445393004 | Right bundle branch block and left posterior fascicular block on electrocardiogram |
| 40482938 | 445309007 | Trifascicular block on electrocardiogram |
| 40482887 | 445263008 | Right bundle branch block and left anterior fascicular block on electrocardiogram |
| 40482505 | 445211001 | Left posterior fascicular block on electrocardiogram |
| 40482086 | 445118002 | Left anterior fascicular block on electrocardiogram |
| 37396252 | 715994002 | 72 hour ambulatory electrocardiographic monitoring |
| 37118682 | 733534002 | Electrocardiographic complete left bundle branch block |
| 37019050 | 713424004 | EKG: focal atrial tachycardia |
| 37017194 | 713427006 | EKG: complete right bundle branch block |
| 37017193 | 713426002 | EKG: Incomplete right bundle branch block |
| 37017191 | 713423005 | EKG: multifocal atrial tachycardia |
| 37017190 | 713422000 | EKG: atrial tachycardia |
| 37017189 | 713421007 | EKG ventricular tachycardia monomorphic |
| 37017188 | 713420008 | EKG ventricular tachycardia polymorphic |
| 4337811 | 87469009 | Left atrial hypertrophy by EKG |
| 4327859 | 428750005 | Nonspecific ST-T abnormality on electrocardiogram |
| 4327042 | 75444003 | Fetal electrocardiogram |
| 4326653 | 428549008 | Secondary ST-T abnormality on electrocardiogram |
| 4324770 | 428803005 | 3 lead electrocardiographic monitoring |
| 4323962 | 70878000 | Electrocardiogram, rhythm |
| 4321877 | 991002 | Wide QRS complex |
| 4314809 | 86688004 | Apexcardiogram with ECG lead |
| 4311995 | 422952004 | Cardiac telemetry |
| 4311809 | 85734009 | Monitoring of ECG, pressure, blood gases and cardiac output |
| 4311428 | 423863005 | Electrical alternation of heart |
| 4306615 | 83370008 | Ventricular hypertrophy by EKG |
| 4305516 | 82281000 | Atrial hypertrophy by EKG |
| 4300288 | 77566008 | Right atrial hypertrophy by electrocardiogram |
| 4299242 | 77867006 | Shortened QT interval |
| 4295129 | 76388001 | ST segment elevation |
| 4286037 | 68611002 | Electrocardiographic recording from artificial pacemaker |
| 4281078 | 66812002 | Flattened T wave |
| 4277091 | 65331008 | Electrocardiogram, intracardiac |
| 4273023 | 365417009 | QT interval - finding |
| 4273022 | 365416000 | ST segment - finding |
| 4273021 | 365415001 | QRS complex - finding |
| 4272343 | 6412007 | Digitalis toxicity by EKG |
| 4267875 | 61721007 | Counterclockwise vectorcardiographic loop |
| 4261794 | 46136006 | Electrocardiogram with exercise test |
| 4261616 | 45076008 | Masters' stress test, two-step |
| 4261488 | 44998009 | Right ventricular ST changes |
| 4253065 | 73999000 | Posterior myocardial infarction on electrocardiogram |
| 4250762 | 73605004 | Electrocardiogram with vectorcardiogram |
| 4247796 | 7326005 | Inferior myocardial infarction on electrocardiogram |
| 4245977 | 59931005 | Inverted T wave |
| 4244435 | 59462000 | Decreased electrocardiogram voltage |
| 4243318 | 38531000 | Diminished LV forces by EKG |
| 4238879 | 69318006 | Shortened ST interval |
| 4237055 | 91096005 | Monitoring of ECG at surgery |
| 4236501 | 91090004 | Monitoring of ECG and pressure during major surgery |
| 4235899 | 90728002 | Intra-atrial recording |
| 4235362 | 438605009 | Measurement of microvolt T wave alternans for ventricular arrhythmia assessment |
| 4225910 | 85090007 | Dextrocardia/situs inversus finding |
| 4225900 | 85038003 | Tracing of carotid pulse with ECG lead |
| 4218204 | 398002 | Left axis deviation greater than -90 degrees by EKG |
| 4215796 | 71869000 | Right ventricular recording |
| 4215406 | 39732003 | Left axis deviation |
| 4211365 | 56743000 | Ventricular inversion |
| 4211020 | 57338000 | Electrocardiogram, esophageal lead |
| 4206924 | 56260006 | Prolonged ST segment |
| 4204712 | 55106003 | Apexcardiography |
| 4195787 | 44325009 | Diminished RV forces by EKG |
| 4187078 | 46825001 | Electrocardiographic monitoring |
| 4184762 | 55930002 | EKG ST segment changes |
| 4184348 | 43630006 | Anterolateral infarction by electrocardiogram |
| 4181994 | 366170001 | QRS Axis - finding |
| 4181448 | 428813002 | Pharmacologic and exercise stress test |
| 4180609 | 429731003 | Anterior myocardial infarction on electrocardiogram |
| 4179374 | 429163003 | 15 lead electrocardiographic monitoring |
| 4171521 | 49578007 | Shortened PR interval |
| 4166218 | 47665007 | Right axis deviation |
| 4163951 | 29303009 | Electrocardiographic procedure |
| 4155644 | 284020009 | EKG ST segment monitoring |
| 4154744 | 284021008 | ECG automated dysrhythmia monitoring |
| 4153511 | 370365005 | EKG: left ventricular strain |
| 4151424 | 3130004 | Monitoring of cardiac output by ECG |
| 4150682 | 31303001 | Electrocardiogram with sub-maximal exercise test |
| 4148302 | 30597000 | Bundle of His recording |
| 4146418 | 427485008 | EKG: asystole |
| 4146297 | 427446001 | 48 hour ambulatory electrocardiographic monitoring |
| 4145916 | 34455003 | Electrocardiogram monitoring, 10 hour portrait |
| 4145510 | 426761007 | EKG: supraventricular tachycardia |
| 4145308 | 268400002 | 12 lead ECG |
| 4144544 | 34405006 | Continuous electrocardiogram |
| 4141663 | 426995002 | EKG: junctional escape rhythm |
| 4141651 | 266706003 | Continuous ECG monitoring |
| 4141527 | 426882006 | EKG: torsades de pointes |
| 4140912 | 425808002 | 18 lead electrocardiographic monitoring |
| 4140676 | 33442008 | Signal-averaged electrocardiography |
| 4140473 | 427047002 | Holter extended electrocardiographic recording |
| 4139185 | 426434006 | EKG: anterior ischemia |
| 4138921 | 426183003 | EKG: Mobitz type II atrioventricular block |
| 4137879 | 425623009 | EKG: lateral ischemia |
| 4137237 | 425529000 | Cardiomemo electrocardiographic monitoring |
| 4137208 | 425419005 | EKG: inferior ischemia |
| 4119098 | 22872007 | Stimulation of carotid sinus with simultaneous electrocardiographic monitoring |
| 4111551 | 195101003 | Wandering atrial pacemaker |
| 4104995 | 29489007 | EKG axis perpendicular to frontal plane |
| 4102780 | 28630001 | Narrow QRS complex |
| 4102533 | 25488008 | Left ventricular hypertrophy by electrocardiogram |
| 4100215 | 27649004 | Left ventricular ST changes |
| 4098508 | 252417001 | 24 Hour ECG |
| 4095285 | 26141007 | ST segment depression |
| 4092036 | 251264001 | Bifid U wave |
| 4092035 | 251262002 | Flat U wave |
| 4092034 | 251259000 | High T-voltage |
| 4092033 | 251257003 | Reverse tick ST segment |
| 4092030 | 251240002 | Inverted U wave |
| 4092027 | 251232008 | S wave height decreased |
| 4092025 | 251228002 | R wave height increased |
| 4092024 | 251225004 | Q wave height increased |
| 4092019 | 251206002 | Shortened P wave |
| 4092016 | 251194000 | P wave right axis deviation |
| 4091456 | 251148003 | Low QRS voltages in the precordial leads |
| 4089486 | 251261009 | TU depression |
| 4089485 | 251258008 | Bifid T wave |
| 4089481 | 251245007 | PR depression |
| 4089479 | 251242005 | Tall U wave |
| 4089478 | 251238007 | Tall tented T wave |
| 4089477 | 251237002 | Tall T wave |
| 4089476 | 251235005 | QRS complex height decreased |
| 4089475 | 251234009 | QRS complex height increased |
| 4089474 | 251229005 | R wave height decreased |
| 4089473 | 251218006 | Shortened U wave |
| 4089471 | 251212007 | Shortened T wave |
| 4089470 | 251209009 | Shortened ST segment |
| 4089467 | 251198002 | Clockwise cardiac rotation |
| 4088997 | 251256007 | Sloping ST segment |
| 4088993 | 251217001 | Prolonged U wave |
| 4088991 | 251215009 | Shortened TU interval |
| 4088990 | 251214008 | Prolonged TU interval |
| 4088526 | 251260005 | TU elevation |
| 4088525 | 251255006 | Sloping PR interval |
| 4088523 | 251244006 | PR elevation |
| 4088522 | 251241003 | Flattened U wave |
| 4088520 | 251231001 | S wave height increased |
| 4088518 | 251226003 | Q wave height decreased |
| 4088517 | 251223006 | Tall P wave |
| 4088516 | 251222001 | Flattened P wave |
| 4088513 | 251211000 | Prolonged T wave |
| 4088511 | 251205003 | Prolonged P wave |
| 4088510 | 251199005 | Counterclockwise cardiac rotation |
| 4088509 | 251197007 | Superior axis |
| 4088500 | 251147008 | Low QRS voltages in the limb leads |
| 4088499 | 251146004 | Low QRS voltages |
| 4088494 | 251117006 | Type B Wolff-Parkinson-White pattern |
| 4088334 | 251118001 | Lown-Ganong-Levine pattern |
| 4088333 | 251116002 | Type A Wolff-Parkinson-White pattern |
| 4067326 | 20329001 | Low ventricular voltage by EKG |
| 4065394 | 164951009 | EKG: QRS complex abnormal |
| 4065391 | 164937009 | EKG: U wave abnormal |
| 4065390 | 164934002 | EKG: T wave abnormal |
| 4065387 | 164918000 | Pathological Q wave |
| 4065386 | 164917005 | EKG: Q wave abnormal |
| 4065385 | 164916001 | ECG: Q wave normal |
| 4065288 | 164890007 | EKG: atrial flutter |
| 4065287 | 164887001 | EKG: supraventricular arrhythmia |
| 4065283 | 164877000 | EKG: right ventricle hypertrophy |
| 4065282 | 164873001 | EKG:left ventricle hypertrophy |
| 4065279 | 164854000 | ECG normal |
| 4065278 | 164850009 | Ambulatory ECG |
| 4065277 | 164847006 | Standard ECG |
| 4064885 | 164947007 | Prolonged PR interval |
| 4064880 | 164926008 | Deep S wave |
| 4064879 | 164921003 | EKG: R wave abnormal |
| 4064876 | 164912004 | EKG: P wave abnormal |
| 4064874 | 164906009 | EKG: complete atrioventricular block |
| 4064872 | 164901004 | EKG: complete sinoatrial block |
| 4064871 | 164895002 | EKG: ventricular tachycardia |
| 4064746 | 165082004 | Exercise tolerance test normal |
| 4064627 | 164956004 | ECG: Q-T interval abnormal |
| 4064626 | 164955000 | ECG: Q-T interval normal |
| 4064621 | 164929001 | ECG: ST interval normal |
| 4064619 | 164925007 | EKG: S wave abnormal |
| 4064618 | 164922005 | Tall R wave |
| 4064615 | 164911006 | EKG: P wave normal |
| 4064614 | 164909002 | EKG: left bundle branch block |
| 4064611 | 164870003 | EKG: subendocardial infarct |
| 4064610 | 164868007 | Anteroseptal infarction on electrocardiogram |
| 4064609 | 164865005 | EKG: myocardial infarction |
| 4064608 | 164851008 | Ambulatory ECG normal |
| 4064487 | 165084003 | Exercise tolerance test abnormal |
| 4064468 | 164950005 | ECG: QRS complex normal |
| 4064467 | 164946003 | EKG: P-R interval abnormal |
| 4064462 | 164933008 | ECG: T wave normal |
| 4064461 | 164930006 | ECG: ST interval abnormal |
| 4064460 | 164907000 | EKG: right bundle branch block |
| 4064459 | 164905008 | Mobitz type I second degree atrioventricular block on electrocardiogram |
| 4064457 | 164898000 | EKG: heart block |
| 4064455 | 164896001 | EKG: ventricular fibrillation |
| 4064453 | 164893009 | EKG: ventricular arrhythmia |
| 4064452 | 164889003 | ECG: atrial fibrillation |
| 4064350 | 164871004 | Lateral infarction on electrocardiogram |
| 4064349 | 164869004 | EKG:posterior/inferior infarct |
| 4064348 | 164867002 | EKG: old myocardial infarction |
| 4064346 | 164861001 | EKG myocardial ischemia |
| 4064345 | 164852001 | Ambulatory ECG abnormal |
| 4053165 | 23638006 | Electrocardiogram with maximal exercise test |
| 4044736 | 129099008 | Ramp protocol |
| 4044735 | 129098000 | Ellestad protocol |
| 4043543 | 129100000 | Pepper protocol |
| 4034869 | 15291005 | Left ventricular recording |
| 4033557 | 14769009 | Tilt table test |
| 4032141 | 14431003 | Electrocardiogram, single lead |
| 4030021 | 1077002 | Septal infarction by electrocardiogram |
| 4028481 | 13478008 | Electrocardiogram, intracardiac, His bundle recording |
| 4020195 | 1155004 | EKG hypertrophy |
| 4018841 | 175131000 | Percutaneous transluminal electrophysiological studies on conducting system of heart |
| 4008859 | 111975006 | Prolonged QT interval |
| 4006458 | 11148001 | Intracardiac electrophysiologic procedure with ECG |
| 2617472 | G0405 | Electrocardiogram, routine ecg with 12 leads; interpretation and report only, performed as a screening for the initial preventive physical examination |
| 2617471 | G0404 | Electrocardiogram, routine ecg with 12 leads; tracing only, without interpretation and report, performed as a screening for the initial preventive physical examination |
| 2617470 | G0403 | Electrocardiogram, routine ecg with 12 leads; performed as a screening for the initial preventive physical examination with interpretation and report |
| 2313972 | 93798 | Physician or other qualified health care professional services for outpatient cardiac rehabilitation; with continuous ECG monitoring (per session) |
| 2313963 | 93745 | Initial set-up and programming by a physician or other qualified health care professional of wearable cardioverter-defibrillator includes initial programming of system, establishing baseline electronic ECG, transmission of data to data repository, patient |
| 2313941 | 93660 | Evaluation of cardiovascular function with tilt table evaluation, with continuous ECG monitoring and intermittent blood pressure monitoring, with or without pharmacological intervention |
| 2313930 | 93621 | Comprehensive electrophysiologic evaluation including insertion and repositioning of multiple electrode catheters with induction or attempted induction of arrhythmia; with left atrial pacing and recording from coronary sinus or left atrium (List separatel |
| 2313929 | 93620 | Comprehensive electrophysiologic evaluation including insertion and repositioning of multiple electrode catheters with induction or attempted induction of arrhythmia; with right atrial pacing and recording, right ventricular pacing and recording, His bund |
| 2313926 | 93616 | Esophageal recording of atrial electrogram with or without ventricular electrogram(s); with pacing |
| 2313925 | 93615 | Esophageal recording of atrial electrogram with or without ventricular electrogram(s) |
| 2313919 | 93603 | Right ventricular recording |
| 2313918 | 93602 | Intra-atrial recording |
| 2313917 | 93600 | Bundle of His recording |
| 2313866 | 93299 | Interrogation device evaluation(s), (remote) up to 30 days; implantable cardiovascular monitor system or implantable loop recorder system, remote data acquisition(s), receipt of transmissions and technician review, technical support and distribution of re |
| 2313865 | 93298 | Interrogation device evaluation(s), (remote) up to 30 days; implantable loop recorder system, including analysis of recorded heart rhythm data, analysis, review(s) and report(s) by a physician or other qualified health care professional |
| 2313858 | 93291 | Interrogation device evaluation (in person) with analysis, review and report by a physician or other qualified health care professional, includes connection, recording and disconnection per patient encounter; implantable loop recorder system, including he |
| 2313852 | 93285 | Programming device evaluation (in person) with iterative adjustment of the implantable device to test the function of the device and select optimal permanent programmed values with analysis, review and report by a physician or other qualified health care |
| 2313845 | 93278 | Signal-averaged electrocardiography (SAECG), with or without ECG |
| 2313844 | 93272 | External patient and, when performed, auto activated electrocardiographic rhythm derived event recording with symptom-related memory loop with remote download capability up to 30 days, 24-hour attended monitoring; review and interpretation by a physician |
| 2313843 | 93271 | External patient and, when performed, auto activated electrocardiographic rhythm derived event recording with symptom-related memory loop with remote download capability up to 30 days, 24-hour attended monitoring; transmission and analysis |
| 2313842 | 93270 | External patient and, when performed, auto activated electrocardiographic rhythm derived event recording with symptom-related memory loop with remote download capability up to 30 days, 24-hour attended monitoring; recording (includes connection, recording |
| 2313841 | 93268 | External patient and, when performed, auto activated electrocardiographic rhythm derived event recording with symptom-related memory loop with remote download capability up to 30 days, 24-hour attended monitoring; includes transmission, review and interpr |
| 2313833 | 93229 | External mobile cardiovascular telemetry with electrocardiographic recording, concurrent computerized real time data analysis and greater than 24 hours of accessible ECG data storage (retrievable with query) with ECG triggered and patient selected events |
| 2313832 | 93228 | External mobile cardiovascular telemetry with electrocardiographic recording, concurrent computerized real time data analysis and greater than 24 hours of accessible ECG data storage (retrievable with query) with ECG triggered and patient selected events |
| 2313831 | 93227 | External electrocardiographic recording up to 48 hours by continuous rhythm recording and storage; review and interpretation by a physician or other qualified health care professional |
| 2313830 | 93226 | External electrocardiographic recording up to 48 hours by continuous rhythm recording and storage; scanning analysis with report |
| 2313829 | 93225 | External electrocardiographic recording up to 48 hours by continuous rhythm recording and storage; recording (includes connection, recording, and disconnection) |
| 2313828 | 93224 | External electrocardiographic recording up to 48 hours by continuous rhythm recording and storage; includes recording, scanning analysis with report, review and interpretation by a physician or other qualified health care professional |
| 2313827 | 93042 | Rhythm ECG, 1-3 leads; interpretation and report only |
| 2313826 | 93041 | Rhythm ECG, 1-3 leads; tracing only without interpretation and report |
| 2313825 | 93040 | Rhythm ECG, 1-3 leads; with interpretation and report |
| 2313824 | 93025 | Microvolt T-wave alternans for assessment of ventricular arrhythmias |
| 2313822 | 93018 | Cardiovascular stress test using maximal or submaximal treadmill or bicycle exercise, continuous electrocardiographic monitoring, and/or pharmacological stress; interpretation and report only |
| 2313821 | 93017 | Cardiovascular stress test using maximal or submaximal treadmill or bicycle exercise, continuous electrocardiographic monitoring, and/or pharmacological stress; tracing only, without interpretation and report |
| 2313820 | 93016 | Cardiovascular stress test using maximal or submaximal treadmill or bicycle exercise, continuous electrocardiographic monitoring, and/or pharmacological stress; supervision only, without interpretation and report |
| 2313819 | 93015 | Cardiovascular stress test using maximal or submaximal treadmill or bicycle exercise, continuous electrocardiographic monitoring, and/or pharmacological stress; with supervision, interpretation and report |
| 2313816 | 93010 | Electrocardiogram, routine ECG with at least 12 leads; interpretation and report only |
| 2313815 | 93005 | Electrocardiogram, routine ECG with at least 12 leads; tracing only, without interpretation and report |
| 2313814 | 93000 | Electrocardiogram, routine ECG with at least 12 leads; with interpretation and report |
| 2106386 | 3120F | 12-Lead ECG Performed (EM) |
| 2106185 | 3020F | Left ventricular function (LVF) assessment (eg, echocardiography, nuclear test, or ventriculography) documented in the medical record (Includes quantitative or qualitative assessment results) (NMA-No Measure Associated) |
| 2101754 | 0180T | Electrocardiogram, 64 leads or greater, with graphic presentation and analysis; interpretation and report only |
| 2101753 | 0179T | Electrocardiogram, 64 leads or greater, with graphic presentation and analysis; tracing and graphics only, without interpretation and report |
| 2101752 | 0178T | Electrocardiogram, 64 leads or greater, with graphic presentation and analysis; with interpretation and report |
| 2007082 | 89.57 | Apexcardiogram (with ECG lead) |
| 2007081 | 89.56 | Carotid pulse tracing with ECG lead |
| 2007079 | 89.54 | Electrographic monitoring |
| 2007077 | 89.52 | Electrocardiogram |
| 2007076 | 89.51 | Rhythm electrocardiogram |
| 2007075 | 89.5 | Ambulatory cardiac monitoring |
| 2007056 | 89.42 | Masters' two-step stress test |
| 320536 | 102594003 | Electrocardiogram abnormal |

Snomed codes defining ischemic or hemorrhagic stroke

| Id | Code | Name |
| --- | --- | --- |
| 46273649 | 3.4181E+13 | Cerebral infarction due to occlusion of basilar artery |
| 46270381 | 2.93831E+14 | Cerebral infarction due to stenosis of precerebral artery |
| 46270380 | 2.93811E+14 | Cerebral infarction due to vertebral artery stenosis |
| 46270031 | 1.25081E+14 | Cerebral infarction due to occlusion of precerebral artery |
| 45772786 | 705128004 | Cerebral infarction due to embolism of middle cerebral artery |
| 45767658 | 705130002 | Cerebral infarction due to thrombosis of middle cerebral artery |
| 44782773 | 3.4191E+13 | Cerebral infarction due to vertebral artery occlusion |
| 43531607 | 9.9451E+13 | Cerebral infarction due to stenosis of carotid artery |
| 43530683 | 1.49821E+14 | Cerebral infarction due to carotid artery occlusion |
| 42535524 | 3.30421E+14 | Lacunar ataxic hemiparesis of right nondominant side |
| 42535523 | 3.30411E+14 | Lacunar ataxic hemiparesis of left nondominant side |
| 42535466 | 2.92861E+14 | Lacunar ataxic hemiparesis of left dominant side |
| 42535465 | 2.92851E+14 | Lacunar ataxic hemiparesis of right dominant side |
| 40479572 | 441526008 | Infarct of cerebrum due to iatrogenic cerebrovascular accident |
| 37395562 | 1.06021E+14 | Multi-infarct dementia due to atherosclerosis |
| 37116473 | 733199002 | Multifocal cerebral infarction due to and following procedure on cardiovascular system |
| 37110679 | 724994008 | Cerebral ischaemic stroke due to stenosis of extracranial large artery |
| 37110678 | 724993002 | Cerebral ischaemic stroke due to occlusion of extracranial large artery |
| 36717605 | 723082006 | Silent cerebral infarct |
| 4319146 | 95830009 | Pituitary infarction |
| 4146185 | 307767006 | Right sided cerebral infarction |
| 4145897 | 307363008 | Multiple lacunar infarcts |
| 4142739 | 427296003 | Thalamic infarction |
| 4141405 | 307766002 | Left sided cerebral infarction |
| 4138327 | 426107000 | Acute lacunar infarction |
| 4131383 | 413102000 | Infarction of basal ganglia |
| 4129534 | 237701005 | Pituitary apoplexy |
| 4119140 | 302904002 | Infarction of visual cortex |
| 4111714 | 195230003 | Cerebral infarction due to cerebral venous thrombosis, non-pyogenic |
| 4110192 | 195189003 | Cerebral infarction due to thrombosis of cerebral arteries |
| 4110190 | 195186005 | Cerebral infarction due to embolism of precerebral arteries |
| 4110189 | 195185009 | Cerebral infarct due to thrombosis of precerebral arteries |
| 4108356 | 195190007 | Cerebral infarction due to embolism of cerebral arteries |
| 4077086 | 276219001 | Occipital cerebral infarction |
| 4048784 | 230707007 | Anterior cerebral circulation hemorrhagic infarction |
| 4046362 | 230706003 | Hemorrhagic cerebral infarction |
| 4046361 | 230701008 | Pure sensorimotor lacunar infarction |
| 4046360 | 230698000 | Lacunar infarction |
| 4046359 | 230695002 | Partial anterior cerebral circulation infarction |
| 4046358 | 230694003 | Total anterior cerebral circulation infarction |
| 4046237 | 230523009 | Infarction of optic radiation |
| 4046090 | 230287006 | Mixed cortical and subcortical vascular dementia |
| 4046089 | 230285003 | Vascular dementia of acute onset |
| 4045741 | 230703006 | Dysarthria-clumsy hand syndrome |
| 4045740 | 230702001 | Lacunar ataxic hemiparesis |
| 4045738 | 230700009 | Pure sensory lacunar infarction |
| 4045737 | 230699008 | Pure motor lacunar infarction |
| 4045735 | 230693009 | Anterior cerebral circulation infarction |
| 4043732 | 230708002 | Posterior cerebral circulation hemorrhagic infarction |
| 4043731 | 230692004 | Infarction - precerebral |
| 4031045 | 14309005 | Anterior choroidal artery syndrome |
| 765515 | 4.34991E+14 | Cerebral infarction due to basilar artery stenosis |
| 763015 | 4.34961E+14 | Cerebral infarction due to middle cerebral artery occlusion |
| 762951 | 4.34151E+14 | Cerebral infarction due to anterior cerebral artery occlusion |
| 762937 | 4.33961E+14 | Cerebral infarction due to cerebral venous thrombosis |
| 762935 | 4.33931E+14 | Cerebral infarction due to internal carotid artery occlusion |
| 762934 | 4.33911E+14 | Cerebral infarction due to posterior cerebral artery occlusion |
| 762933 | 4.33891E+14 | Cerebral infarction due to cerebral artery occlusion |
| 761110 | 1.51161E+14 | Bilateral cerebral infarction due to precererbral arterial occlusion |
| 444091 | 10349009 | Multi-infarct dementia with delirium |
| 443864 | 14070001 | Multi-infarct dementia with depression |
| 443790 | 25772007 | Multi-infarct dementia with delusions |
| 443454 | 432504007 | Cerebral infarction |
| 441874 | 71444005 | Cerebral thrombosis |
| 439847 | 1386000 | Intracranial hemorrhage |
| 432923 | 21454007 | Subarachnoid hemorrhage |
| 379778 | 56267009 | Multi-infarct dementia |
| 377254 | 70936005 | Multi-infarct dementia, uncomplicated |
| 376713 | 274100004 | Cerebral hemorrhage |
| 375557 | 75543006 | Cerebral embolism |
| 372924 | 20059004 | Cerebral artery occlusion |

Anticoagulants RxNorm ingredients:

| Id | Code | Name |
| --- | --- | --- |
| 1112807 | 1191 | Aspirin |
| 1301025 | 67108 | Enoxaparin |
| 1310149 | 11289 | Warfarin |
| 1367571 | 5224 | heparin |
| 1322184 | 32968 | clopidogrel |
| 40241331 | 1114195 | rivaroxaban |
| 43013024 | 1364430 | apixaban |
| 977968 | 56466 | sodium citrate |
| 1331270 | 3521 | Dipyridamole |
| 1350310 | 21107 | cilostazol |
| 40228152 | 1037042 | dabigatran etexilate |
| 40163718 | 613391 | prasugrel |
| 40241186 | 1116632 | Ticagrelor |
| 1322199 | 75635 | eptifibatide |
| 1301065 | 67109 | Dalteparin |
| 19084670 | 60819 | bivalirudin |
| 19035344 | 8150 | Phenprocoumon |
| 1315865 | 321208 | fondaparinux |
| 1302398 | 10594 | Ticlopidine |
| 19047423 | 83929 | abciximab |
| 1308473 | 69646 | tinzaparin |
| 45892847 | 1599538 | edoxaban |
| 19113013 | 50097 | fluindione |
| 1322207 | 15202 | argatroban |
| 1354118 | 8814 | Epoprostenol |
| 1327256 | 343048 | Treprostinil |
| 19001014 | 67031 | Nadroparin |
| 19017067 | 73137 | tirofiban |
| 42801108 | 8834 | Protein C |
| 19098548 | 259280 | Tenecteplase |
| 1436169 | 1009 | Antithrombin III |
| 19129274 | 75960 | reviparin |
| 35604848 | 1729002 | selexipag |
| 19024063 | 154 | Acenocoumarol |
| 1344992 | 40138 | Iloprost |
| 44818499 | 1537034 | vorapaxar |
| 19024191 | 76895 | Reteplase |
| 1307515 | 11055 | Urokinase |
| 46275677 | 1656052 | cangrelor |
| 19077421 | 5556 | Hymecromone |
| 19026343 | 78484 | danaparoid |
| 19008276 | 69528 | parnaparin |
| 19092139 | 237057 | lepirudin |
| 19136187 | 10106 | Streptokinase |
| 1731597 | 352374 | drotrecogin alfa |
| 954696 | 4388 | Plasmin |
| 42898933 | 1311089 | defibrotide |
| 19044890 | 40028 | Anistreplase |
| 19011712 | 114934 | desirudin |
| 19069137 | 280611 | bemiparin |
| 19069107 | 27518 | indobufen |
| 19053746 | 87866 | ardeparin |
| 19042778 | 38655 | triflusal |
| 19033934 | 8130 | Phenindione |
| 19024544 | 772 | Ancrod |
| 19018364 | 163426 | tioclomarol |
| 1332661 | 17941 | anisindione |
| 1325124 | 1598 | Dicumarol |
