# Supplementary material for: Feasibility and evaluation of a large-scale external validation approach for patient-level prediction in an international data network: validation of models predicting stroke in female patients newly diagnosed with atrial fibrillation
Source: BMC Med Res Methodol. 2020 May 6;20:102. doi: 10.1186/s12874-020-00991-3 (PMC7201646; doi:10.1186/s12874-020-00991-3)
Supplement: Supplementary file 3 — Additional file 3. Appendix C. Characterization of patients. [file 12874_2020_991_MOESM3_ESM.docx]

**APPENDIX C**

Characterization of patients

|  | Fraction of patients with stroke in 1-year who had the covariate | | | | | | | Fraction of patients with no stroke in 1-year who had the covariate | | | | | | |
| --- | --- | --- | --- | --- | --- | --- | --- | --- | --- | --- | --- | --- | --- | --- |
| Covariate | CCAE | MDCD | MDCR | Optum claims | Optum EHR | CUMC | STRIDE | CCAE | MDCD | MDCR | Optum claims | Optum EHR | CUMC | STRIDE |
| Age 65-74 | 0.03 | 0.18 | 0.16 | 0.17 | 0.16 | 0.18 | 0.37 | 0.00 | 0.21 | 0.31 | 0.25 | 0.24 | 0.26 | 0.29 |
| Age 75-84 | 0.00 | 0.30 | 0.39 | 0.51 | 0.54 | 0.36 | 0.26 | 0.00 | 0.31 | 0.42 | 0.41 | 0.43 | 0.29 | 0.33 |
| Age >=85 | 0.00 | 0.35 | 0.44 | 0.25 | 0.20 | 0.31 | 0.18 | 0.00 | 0.24 | 0.27 | 0.12 | 0.11 | 0.16 | 0.13 |
| CHF | 0.19 | 0.50 | 0.31 | 0.35 | 0.26 | 0.38 | - | 0.08 | 0.41 | 0.21 | 0.23 | 0.15 | 0.21 | - |
| Chronic renal disease | 0.11 | 0.26 | 0.13 | 0.23 | 0.19 | 0.12 | - | 0.03 | 0.20 | 0.09 | 0.16 | 0.10 | 0.06 | - |
| Congestive cardiac failure | 0.20 | 0.50 | 0.32 | 0.36 | 0.26 | 0.40 | - | 0.08 | 0.41 | 0.22 | 0.24 | 0.15 | 0.23 | - |
| Coronary heart disease | 0.17 | 0.37 | 0.28 | 0.31 | 0.22 | 0.17 | - | 0.11 | 0.34 | 0.25 | 0.24 | 0.16 | 0.18 | - |
| diabetes | 0.31 | 0.46 | 0.26 | 0.32 | 0.24 | 0.22 | 0.18 | 0.19 | 0.44 | 0.22 | 0.27 | 0.19 | 0.17 | 0.10 |
| eGFR<45 or ESRD | 0.03 | 0.05 | 0.01 | 0.02 | 0.02 | - | - | 0.01 | 0.03 | 0.01 | 0.01 | 0.01 | - | - |
| Former smoker | 0.13 | 0.14 | 0.03 | 0.08 | 0.05 | - | - | 0.07 | 0.16 | 0.03 | 0.08 | 0.05 | - | - |
| Hypertension | 0.65 | 0.89 | 0.81 | 0.87 | 0.72 | 0.60 | 0.35 | 0.55 | 0.87 | 0.76 | 0.78 | 0.60 | 0.55 | 0.46 |
| proteinuria | 0.05 | 0.02 | 0.01 | 0.03 | 0.02 | - | - | 0.02 | 0.02 | 0.01 | 0.03 | 0.01 | - | - |
| Rheumatoid arthritis | 0.06 | 0.05 | 0.05 | 0.06 | 0.03 | 0.04 | - | 0.03 | 0.05 | 0.04 | 0.05 | 0.03 | 0.03 | - |
| Treated hypertension | 0.04 | 0.03 | 0.05 | 0.04 | 0.02 | - | - | 0.01 | 0.01 | 0.03 | 0.03 | 0.02 | - | - |
| Valvular heart disease | 0.29 | 0.35 | 0.32 | 0.36 | 0.18 | 0.44 | 0.25 | 0.24 | 0.31 | 0.30 | 0.33 | 0.17 | 0.38 | 0.18 |

Table 6 – all counts in the AUSOM data were <10 for patients with stroke due to the low number of patients with stroke and are therefore not included in this table. Cells corresponding to counts < 10 are represented by -.
