# Supplementary material for: Feasibility and evaluation of a large-scale external validation approach for patient-level prediction in an international data network: validation of models predicting stroke in female patients newly diagnosed with atrial fibrillation
Source: BMC Med Res Methodol. 2020 May 6;20:102. doi: 10.1186/s12874-020-00991-3 (PMC7201646; doi:10.1186/s12874-020-00991-3)
Supplement: Supplementary file 4 — Additional file 4. Appendix D. Calibration plots for the females of any age target population. [file 12874_2020_991_MOESM4_ESM.docx]

**APPENDIX D**

**Calibration plots for the females of any age target population**


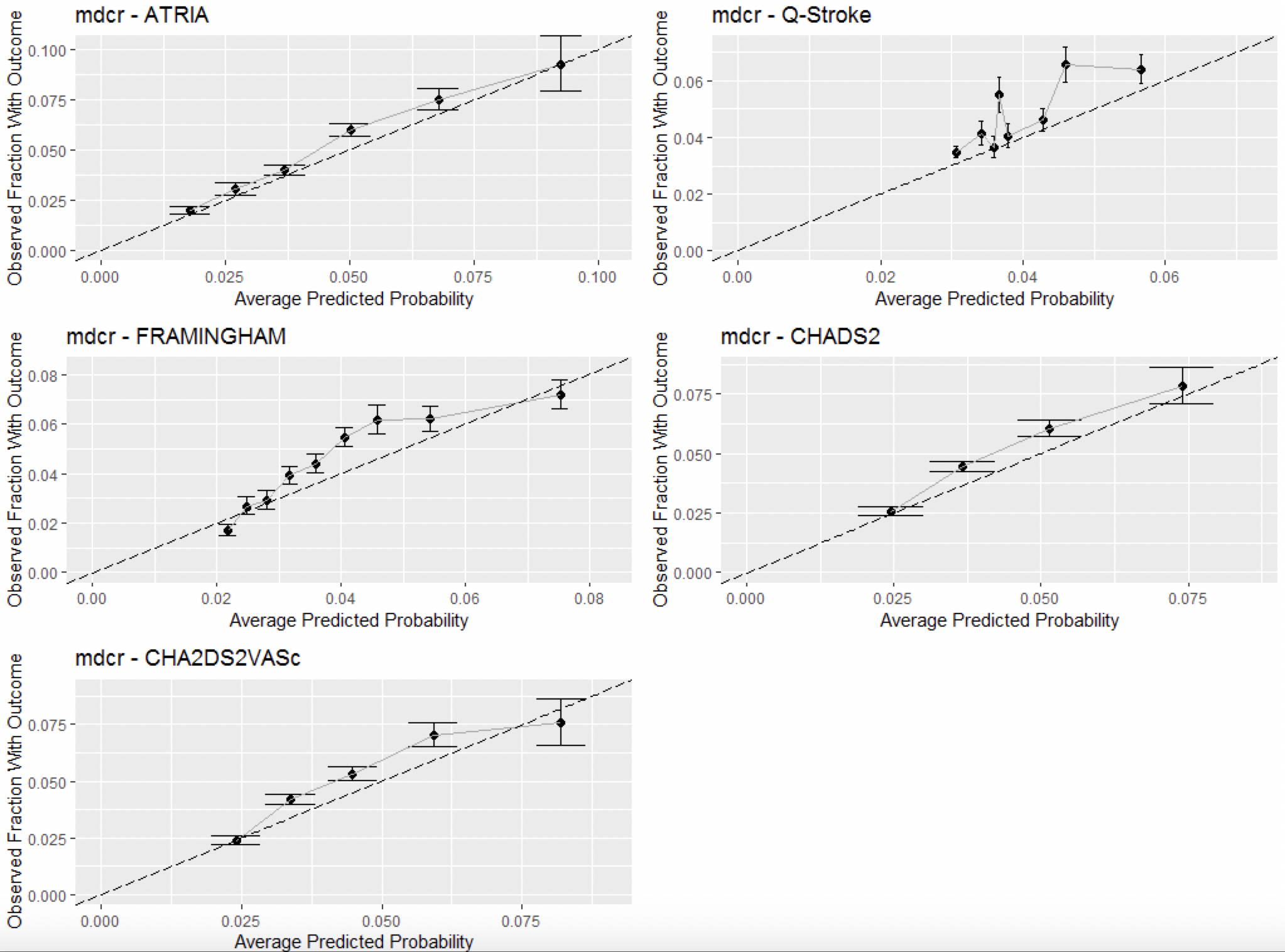


Figure 1- Calibration plots of the models applied to MDCR claims data after recalibrating


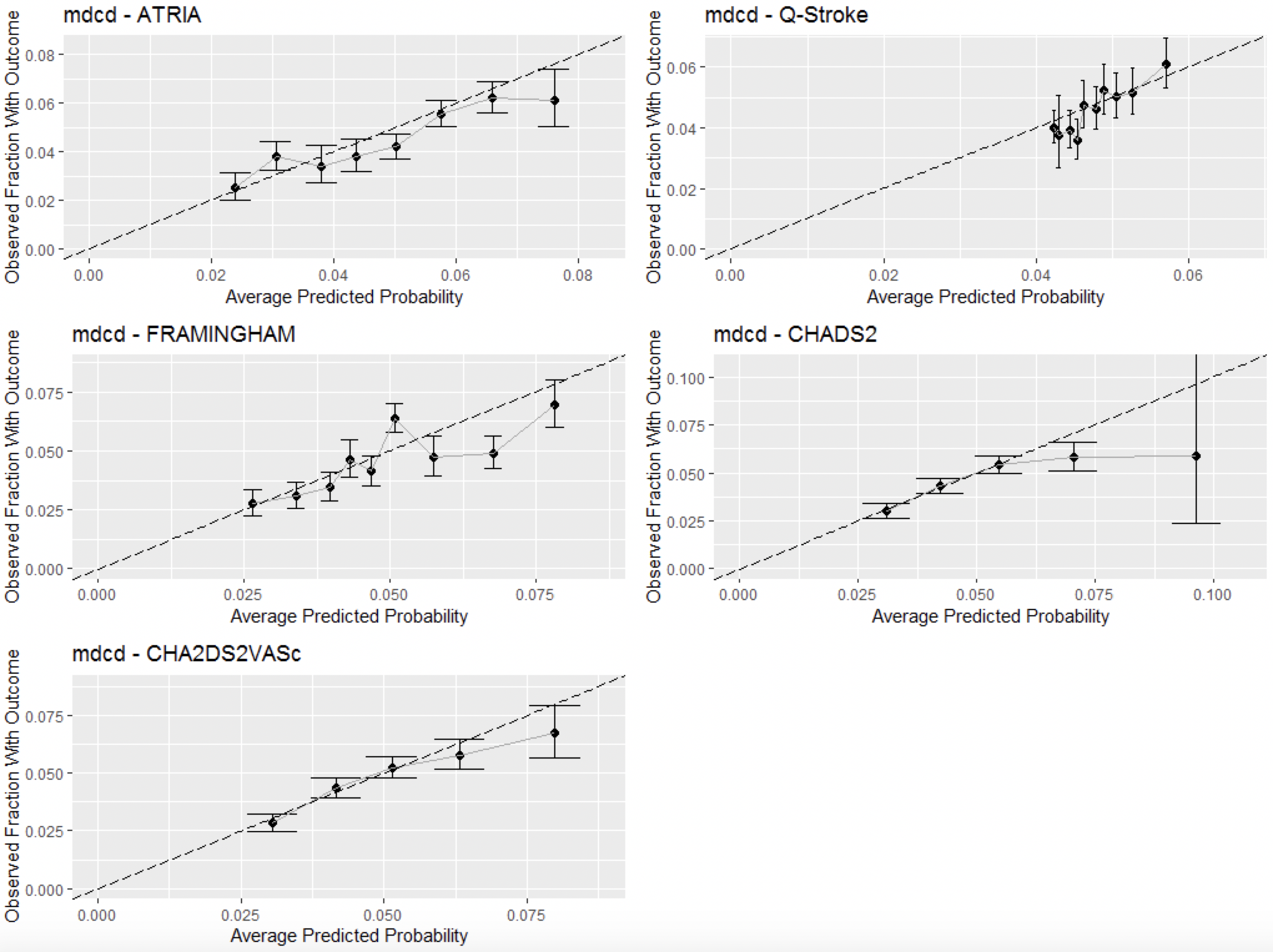


Figure 2- Calibration plots of the models applied to MDCD claims data after recalibrating


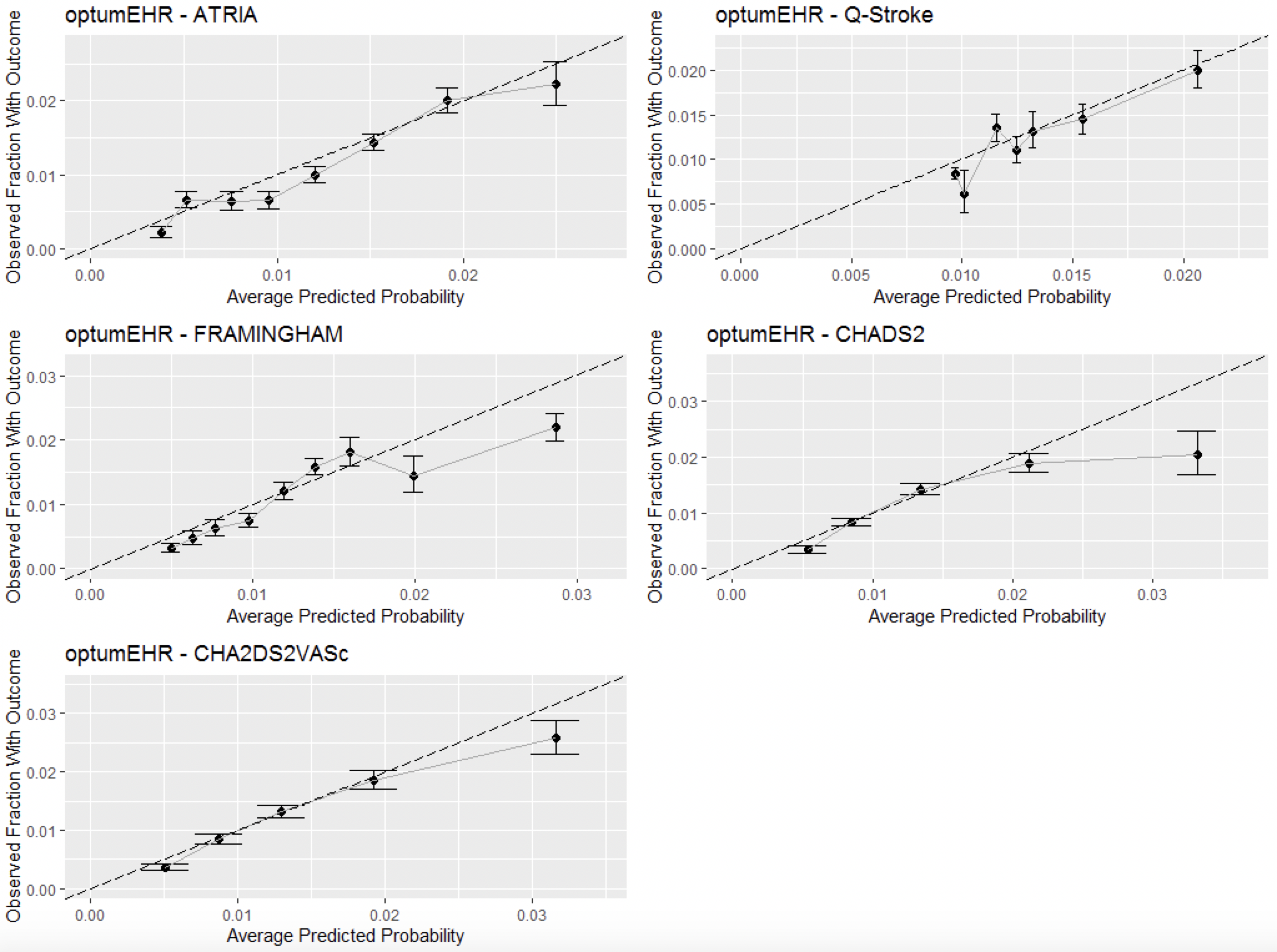


Figure 3- Calibration plots of the models applied to Optum EHR data after recalibrating


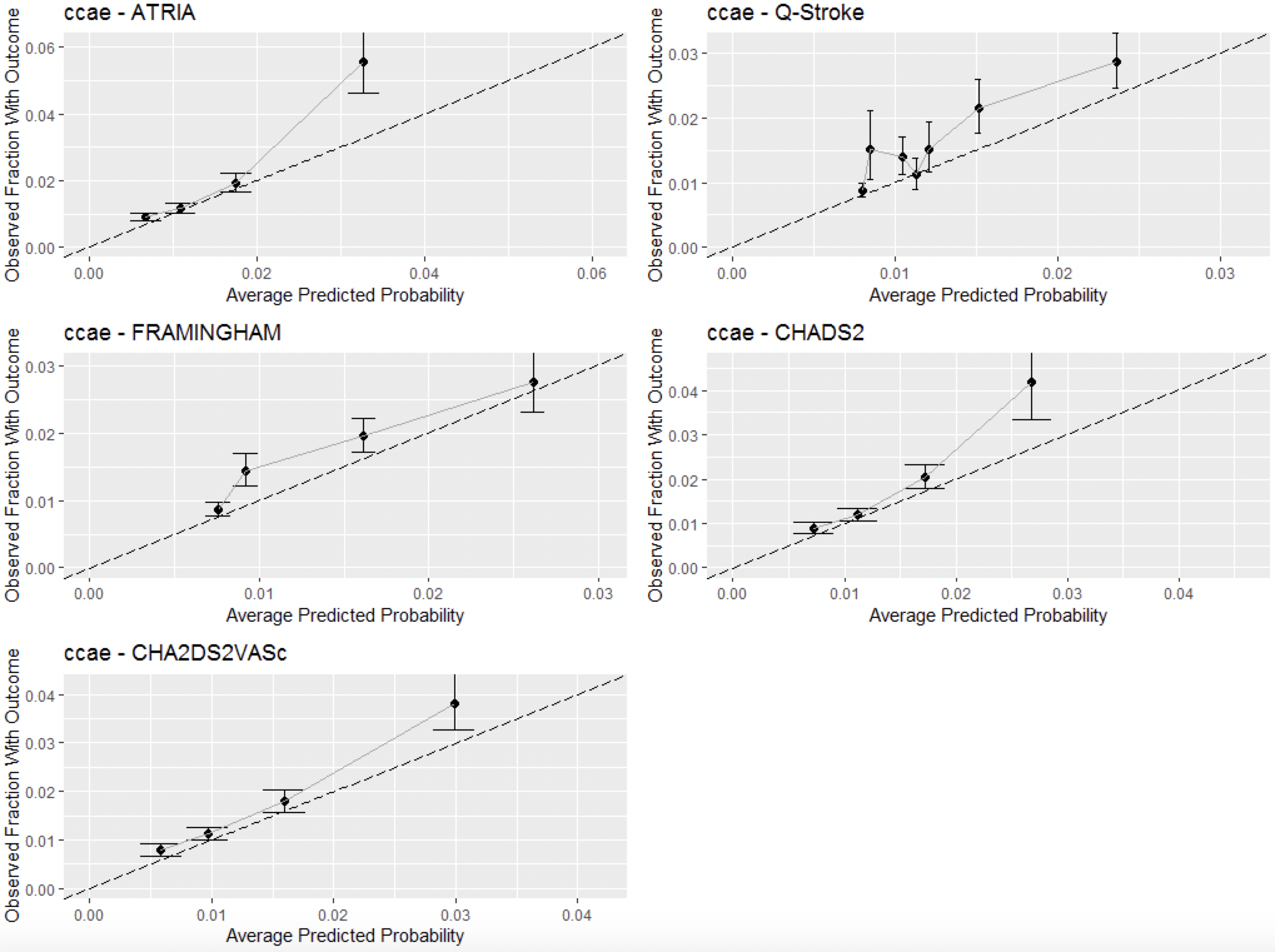


Figure 4- Calibration plots of the models applied to CCAE claims data after recalibrating


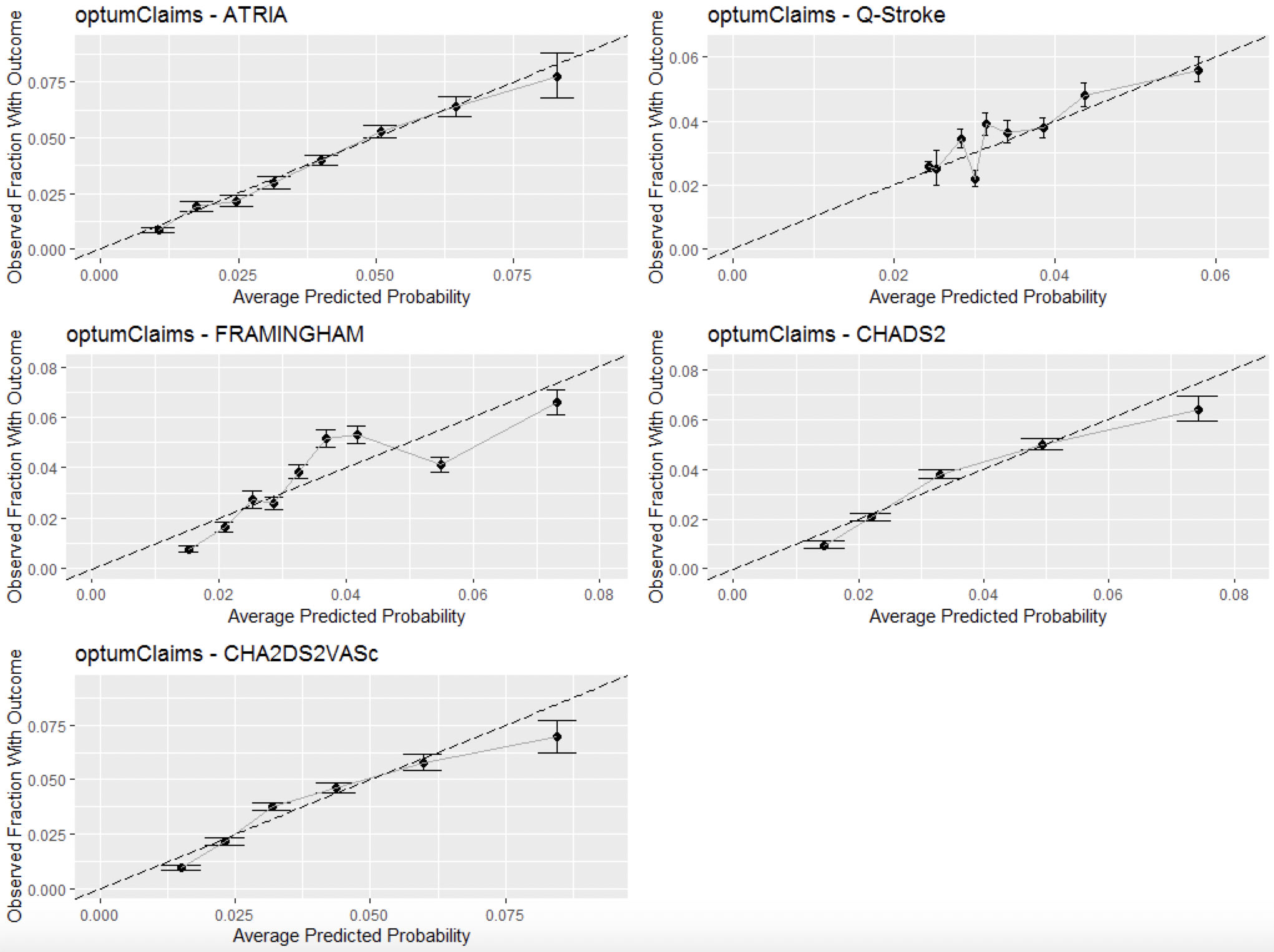


Figure 5- Calibration plots of the models applied to Optum claims data after recalibrating


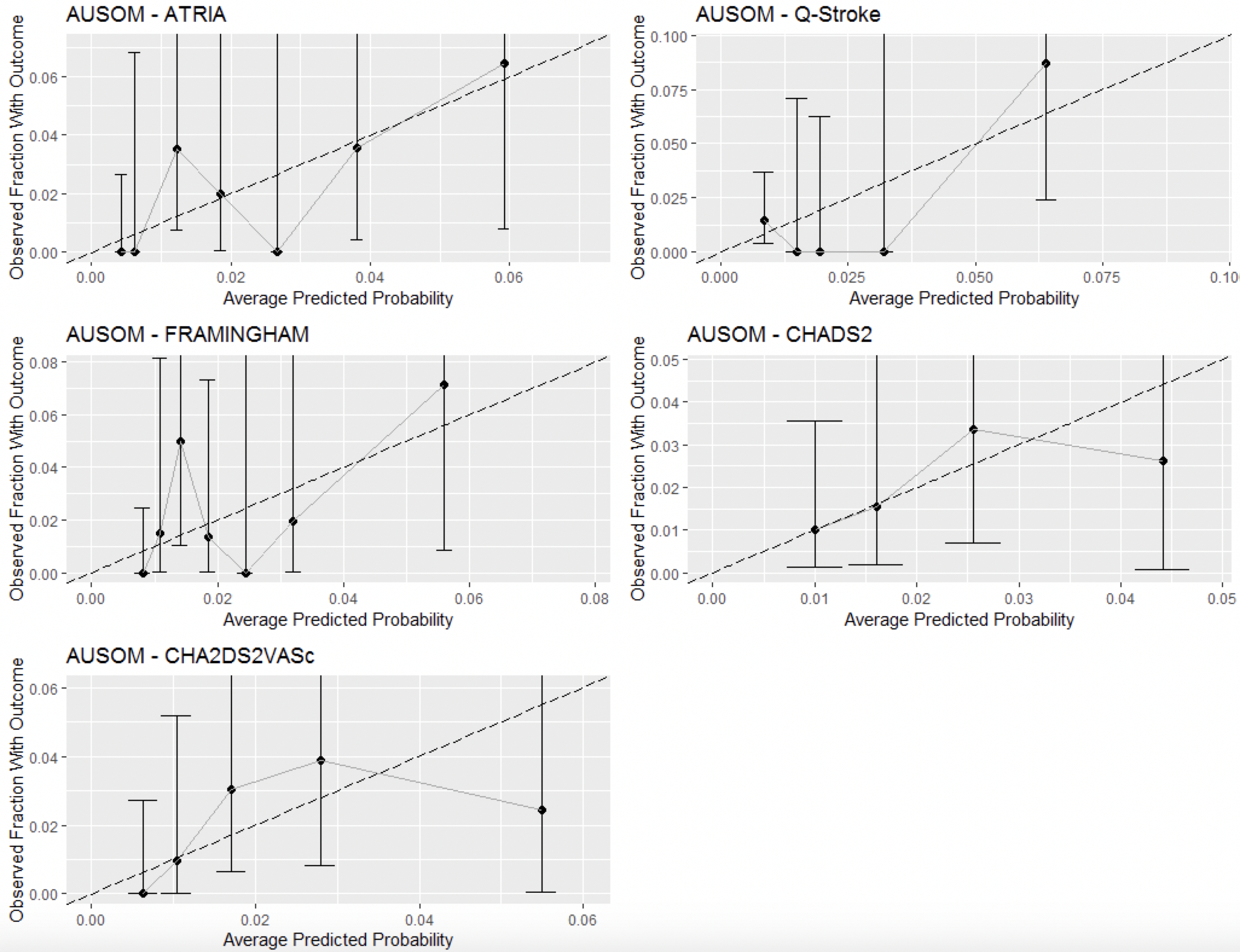


Figure 6 - Calibration plots of the models applied to AUSOM data after recalibrating


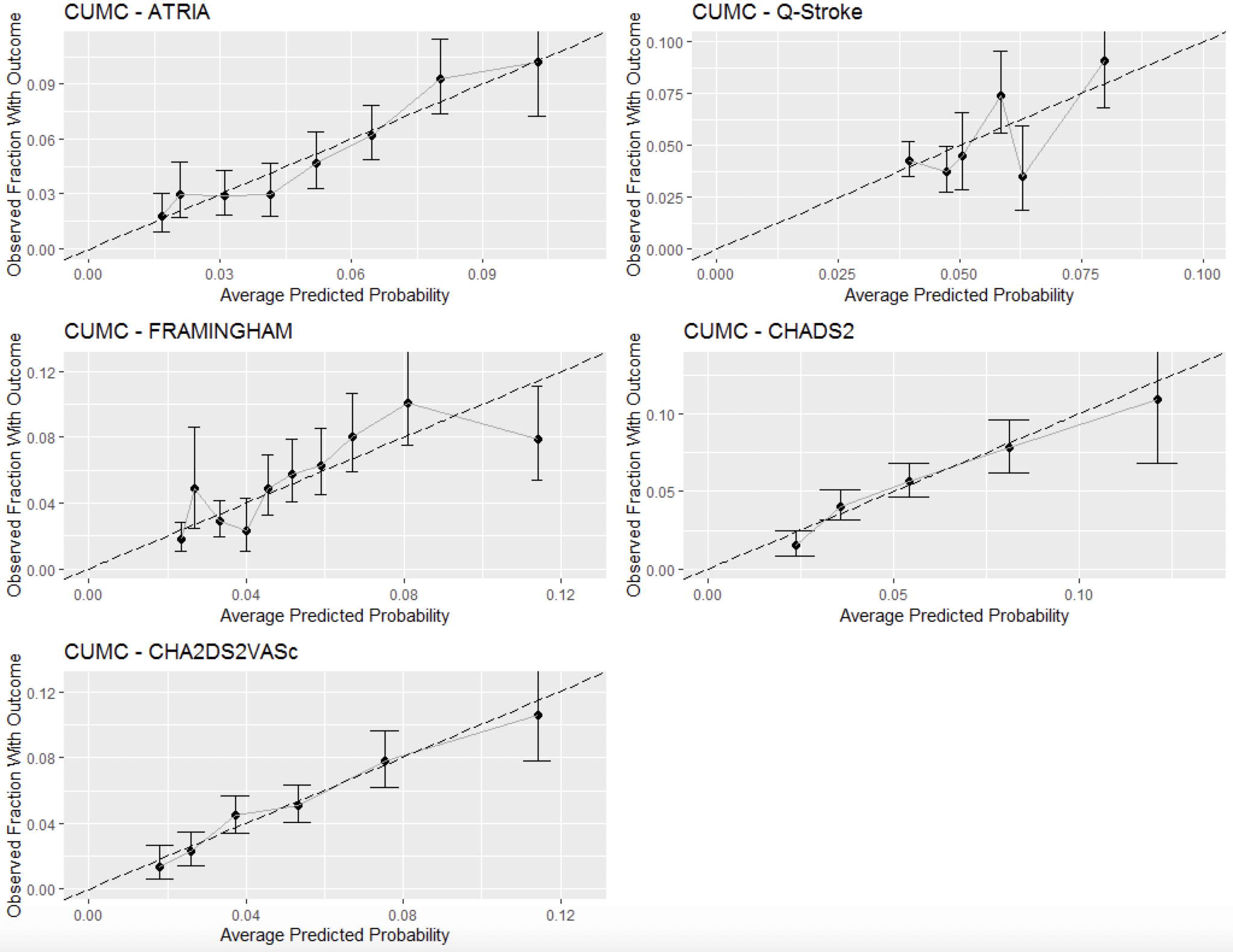


Figure 7 Calibration plots of the models applied to CUMC data after recalibrating

Figure 8 Calibration plots of the models applied to STRIDE data after recalibrating

The recalibration intercept and gradient (when recalibrating using a linear model where x is the total model score and y is the outcome class) for the target population of any age female.

Table 7 - The recalibration intercept and gradient values per database and model

| Database | Model | Intercept | Gradient |
| --- | --- | --- | --- |
| Optum Claims | ATRIA | -4.93 | 0.25 |
|  | Q-stroke | -3.96 | 0.56 |
|  | Framingham | -4.95 | 0.13 |
|  | CHADS_2_ | -4.22 | 0.42 |
|  | CHA_2_DS_2_VASc | -4.74 | 0.33 |
| Optum EHR | ATRIA | -5.81 | 0.23 |
|  | Q-stroke | -4.93 | 0.63 |
|  | Framingham | -6.19 | 0.15 |
|  | CHADS_2_ | -5.22 | 0.46 |
|  | CHA_2_DS_2_VASc | -5.95 | 0.40 |
| CCAE | ATRIA | -5.48 | 0.48 |
|  | Q-stroke | -5.26 | 0.90 |
|  | Framingham | -6.03 | 0.19 |
|  | CHADS_2_ | -4.92 | 0.44 |
|  | CHA_2_DS_2_VASc | -5.65 | 0.51 |
| MDCD | ATRIA | -3.96 | 0.15 |
|  | Q-stroke | -3.19 | 0.17 |
|  | Framingham | -4.13 | 0.09 |
|  | CHADS_2_ | -3.66 | 0.27 |
|  | CHA_2_DS_2_VASc | -4.02 | 0.22 |
| MDCR | ATRIA | -5.51 | 0.32 |
|  | Q-stroke | -3.66 | 0.43 |
|  | Framingham | -4.95 | 0.13 |
|  | CHADS_2_ | -3.97 | 0.35 |
|  | CHA_2_DS_2_VASc | -4.53 | 0.29 |
| AUSOM | ATRIA | -5.82 | 0.37 |
|  | Q-stroke | -5.80 | 2.11 |
|  | Framingham | -5.93 | 0.19 |
|  | CHADS_2_ | -4.59 | 0.48 |
|  | CHA_2_DS_2_VASc | -5.57 | 0.50 |
| CUMC | ATRIA | -4.32 | 0.23 |
|  | Q-stroke | -3.45 | 0.55 |
|  | Framingham | -4.55 | 0.14 |
|  | CHADS_2_ | -3.73 | 0.43 |
|  | CHA_2_DS_2_VASc | -4.37 | 0.37 |
| STRIDE | ATRIA | -4.52 | 0.04 |
|  | Q-stroke | -4.55 | 0.31 |
|  | Framingham | -5.70 | 0.12 |
|  | CHADS_2_ | -4.36 | 0.01 |
|  | CHA_2_DS_2_VASc | -4.89 | 0.17 |
